# Interleukin-6 produces behavioral deficits in pre-pubescent mice independent of neuroinflammation

**DOI:** 10.1016/j.bbi.2025.02.009

**Published:** 2025-02-19

**Authors:** Fernando Janczur Velloso, Rebecca Zaritsky, Rouba Y. Houbeika, Nicolas Rios, Steven W. Levison

**Affiliations:** Department of Pharmacology, Physiology & Neuroscience, New Jersey Medical School, Rutgers University, Newark, NJ 07103, USA

**Keywords:** Cytokines, Chemokines, Maternal infection, Sociability, Communication, Mice

## Abstract

Maternal inflammation during pregnancy increases the offspring’s risk of developing autism, ADHD, schizophrenia, and depression. Epidemiologic studies have demonstrated that maternal infections stimulate the production of interleukin-6 (IL-6), which can cross the placenta and fetal blood–brain barrier to alter brain development with functional and behavioral consequences. To model the effects of increased IL-6 between weeks 24–30 of human gestation, we injected male and female mice with 75 ng IL-6 twice daily, from P3 to P6. Our published studies have shown that this increases circulating IL-6 two-fold, alters post-pubescent ultrasonic vocalization patterns, reduces sociability, and increases self-grooming.

However, most neurodevelopmental disorders in humans manifest in children as young as 2 years of age. Hence, a critical unexplored question is whether behavioral changes in immune activation models can be detected in pre-pubescent mice. Therefore, we evaluated early communication, sociability, and repetitive behaviors in pre-pubescent mice following the IL-6 treatment. A second open question is whether the cellular and behavioral changes are secondary to systemic or neuroinflammation. To address this question, we profiled 18 cytokines and chemokines in the circulation and CNS and evaluated eight immune cell types in P7 male and female brains following systemic IL-6 administration. We found an increase in ultrasonic vocalizations with simpler morphologies produced by the IL-6-injected male pups and a decrease in frequency in the female vocalizations upon removal from the nest at P7. The IL-6-treated male pups also socially interacted less when introduced to a novel mouse vs. controls as juveniles and spent almost twice as much time grooming themselves, a phenotype not present in the females. Tactile sensitivity was also increased, but only in the IL-6-treated female mice. The IL-6-treated mice had increased circulating IL-6 and IL-7 and reduced IL-13 at P7 that were no longer elevated at P14. There were no changes in brain levels of IL-6, IL-10, IL-13 or IL-17A mRNAs at P7. Altogether, these studies show that changes in the three core behavioral domains associated with several psychiatric disorders can be detected early in pre-pubescent mice following a transient developmental increase in IL-6. Yet, these behavioral alterations do not require neuroinflammation.

## 1. Introduction

Environmental factors that produce inflammatory states during pregnancy are intimately linked to neurodevelopmental disorders in the offspring (Bear and Wu, 2016; Carter and Blizard, 2016; Jiang et al., 2016; Mednick et al., 1988; Zerbo et al., 2015). In particular, epidemiological studies have implicated the pro-inflammatory cytokine interleukin-6 (IL-6) as a central hub cytokine that links immune activation to behaviors relevant to Attention Deficit Hyperactivity Disorder (Misiak et al., 2022), Tourette syndrome (Li et al., 2022), Schizophrenia (Patel et al., 2022), and Autism Spectrum Disorder (ASD) (Amgalan et al., 2021; Han et al., 2021; Rudolph et al., 2018; Smith et al., 2007; Spann et al., 2018). Indeed, elevated levels of IL-6 in the amniotic fluid are associated with a later diagnosis of psychiatric disorders (Abdallah et al., 2013), and high levels of IL-6 in mid-gestational maternal serum are associated with ASD with intellectual disabilities (Jones et al., 2017). Studies have demonstrated that maternal IL-6 crosses the placenta into the fetal circulation, where it is then transported across the blood–brain barrier using a saturable transporter that is present in the endothelial cells (Aaltonen et al., 2005; Banks et al., 1994; Dahlgren et al., 2006; Threlkeld et al., 2010; Zaretsky et al., 2004).

Abnormally high levels of IL-6 alter brain development with functional consequences. For example, changes in functional brain connectivity and deficits in working memory and cognition are associated with increased levels of IL-6 in the maternal circulation (Rudolph et al., 2018; Spann et al., 2018). Elevated maternal IL-6 also correlates with changes in the offspring’s amygdala volume and connectivity and with deficits in impulse control at 24 months (Graham et al., 2018). In addition, white matter dysmaturation also has been observed in infants born to mothers who had elevated levels of IL-6 during pregnancy (Rasmussen et al., 2019) and these infants had cognitive deficits that were evident by the end of the first year of postnatal life.

Rodents have modeled the outcomes of activating the maternal immune system on the progeny by employing bacterial lipopolysaccharide (LPS) or viral mimetics (Polyinosinic:polycytidylic acid (Poly(I:C)) (Bilbo et al., 2018). Maternal treatment with these mimetics induces the production of a series of proinflammatory cytokines and leads to behavioral outcomes in the offspring encompassing sociability, communication, and repetitive stereotyped behaviors (Hida et al., 2014; Hsiao et al., 2012; Malkova et al., 2012). In a classic study, Smith et al., 2007 injected poly(I:C) at midgestation into pregnant mice and evaluated the effects of blocking IL-1β, IL-6 and IFNƔ. Only antagonizing IL-6 attenuated the behavioral abnormalities observed in the offspring. Furthermore, a single injection of IL-6 at midgestation was sufficient to induce behavioral abnormalities in the offspring, while IL-1α and TNFα were without effect (Smith et al., 2007). Similarly, sociability deficits induced by LPS were attenuated by controlling plasma IL-6 levels postnatally with the anti-inflammatory drug Pioglitazone (Kirsten et al., 2018).

To establish how increased levels of IL-6 alone will alter brain development during a stage in mouse development corresponding to weeks 24–30 of human gestation, we injected 75 ng of recombinant murine IL-6 into mouse pups between day 3 and 6 postnatally. We previously showed that the two-fold increase in circulating IL-6 produced was sufficient to generate abnormal social behaviors, increased stereotyped behaviors, and communication deficits when the mice were analyzed post-pubescence (Velloso et al., 2022b).

However, behavior develops throughout infancy, conditioned by rapid brain development and contextual stimuli. Therefore, aspects of behavior, like sociability, are expressed differently from infancy to adolescence. Furthermore, most neurodevelopmental disorders in humans present in children as young as 2 years (American Psychiatric Association, 2013); therefore, an essential unexplored question is whether behavioral changes in immune activation models can be detected in pre-pubescent mice. To address this knowledge gap here, we have evaluated communication, sociability, and repetitive behaviors in pre-pubescent mice. Another open question in the literature is whether the cellular changes in the brain and resulting behavioral changes are secondary to persistent systemic or neuroinflammatory states. To address this question, here we have profiled a large panel of cytokines and chemokines and evaluated immune cells in both the circulation and in the CNS acutely after injecting IL-6 into newborn mice.

## 2. Methods

### 2.1. Mice and IL-6 administration regimen

Swiss Webster timed-pregnant females were purchased from Charles River Laboratories. They arrived at gestational day 16 and were maintained in the Comparative Medicine Resources animal facility at Rutgers Biomedical Health Sciences. Animals were maintained in same-sex cages under a 12 h light/dark cycle with food and water available ad libitum. Litters were culled to 12 mice and weaned at P21. All experiments were performed per the approved Rutgers University IACUC protocol #999901108, which followed the National Institutes of Health Guide for the Care and Use of Laboratory Animals (NIH publication No. 80-23). Two to three mice per sex per experimental group were randomly selected from each litter and equally assigned to experimental groups (IL-6 and control). Each group contained mice from at least three litters. The interleukin-6 (IL-6) injection regimen was followed as described previously (Velloso et al., 2022b). In short, mice received two intraperitoneal injections daily from postnatal day 3 (P3) to P5, 6 h apart, and one additional injection at postnatal day 6 (P6). Each injection had a volume of 5 μL of sterile PBS (controls) or PBS containing 75 ng of carrier-free recombinant mouse interleukin-6 (rmIL6, R&D Systems, Minneapolis, MN). Behavior, molecular, and cellular measurements followed from P7 to P21. Body mass and internal temperature (measured with a rectal probe) were measured daily starting at P7 for two weeks and then weekly until P56. Terminal procedures that required blood and brain sample collection were performed on mice that underwent ultrasonic vocalization call recordings. The remaining behavioral tasks and physiological measurements were performed using separate cohorts.

### 2.2. Ultrasonic vocalization calls

Ultrasonic vocalizations (USV) were recorded as a parameter of communication skills. Following a one-hour acclimation, P7 mice were individually isolated from the nest and placed in a clean cage without bedding in a soundproof room. USV calls were recorded for 5 min using an overhead-mounted (30 cm high) high-quality condenser microphone connected to an Avisoft-UltraSoundGate amplifier (116H). Acoustic data were recorded with a sampling rate of 250,000 Hz in the 16-bit format in Avisoft RECORDER and analyzed in SASlab pro software (Avisoft Bioacoustics, Nordbahn, Germany). Parameters measured included the number of ultrasonic calls, interval between calls, average frequency, bandwidth, amplitude, and duration of calls. Further analyses were performed using DeepSqueak (Coffey et al., 2019), including the total number of ultrasonic calls produced, USV physical characteristics, USV morphology classification, and unsupervised UMAP clustering. USVs were classified into 9 clusters based on morphology, using supervised classification after training the classification network using our USV recordings.

### 2.3. Pup retrieval

Pairs of sex-matched P7 IL-6 and PBS-treated mice were removed from the nest and placed on opposite sides of the home cage, opposite to the nest, which was empty before the start of each trial. The mother was placed in the nest and allowed to retrieve the pups. The frequency of the maternal first choice for pup retrieval and the total latency to return the pup to the nest were recorded. Experiments were performed in the same room where the mice were housed.

### 2.4. Reciprocal juvenile social interaction

Reciprocal social interactions were scored for P21 male mice. Briefly, after one hour of acclimation in single-housed cages in the test room, a pair of strain, sex, and age-matched mice socially unfamiliar to each other were simultaneously placed into a neutral, clean (fresh bedding) arena (25 x 35 cm) and allowed to freely interact for 5 min. IL-6-treated mice were paired with both PBS injected (control) and other unfamiliar IL-6-treated mice in separate interaction sessions. Control mice were always paired with different controls. After the session, mice were returned to home cages, and the arena was sanitized with 70 % ethanol. An overhead camera recorded sessions to allow for posterior scoring by multiple trained observers blinded to the treatment groups. Parameters of social behaviors included nose-to-nose sniffing, nose-to-anogenital sniffing, approaching from the front, following, pushing past, and crawling over, as described previously (McFarlane et al., 2008; Silverman et al., 2010; Yang et al., 2012). All behaviors were analyzed for frequency of occurrence.

### 2.5. Self-grooming

Mice were assessed for spontaneous self-grooming as a measure of repetitive behavior. Following a one-hour acclimation in the test room, mice were placed in the test arena, consisting of a cage with fresh bedding. After a 5-minute habituation, mice were recorded over a 10-minute test session by a lateral-mounted camera and later scored for cumulative self-grooming time by a trained observer who remained blinded to the experimental group.

### 2.6. Mechanosensitivity

Mechanosensitivity was evaluated in mice on P13, using a paradigm that measures the frequency of hindpaw withdrawal elicited by stimuli with Von Frey filaments. Following a one-hour acclimation, mice were placed individually in a wire mesh surface immobilized by a plexiglass box. They received 10 pricks in the plantar surface of each hind paw using a 0.04 g Von Frey monofilament. The frequency of paw withdraws was recorded for each hind paw, and the left and right paws were averaged for each animal. The optimal bending strength of the filament was determined previously in animals of the same age.

### 2.7. Cytokine levels

Circulating and cerebral cytokine and chemokine levels (pg/mL) were measured at P7 and P14 in the plasma and brain lysates of PBS and IL-6-treated mice. Plasma was generated by drawing blood by cardiac puncture and centrifuging for 16,000 x g for 15 min at 4 °C in the presence of 6 U/mL of Heparin. Mice were *trans*-cardiac perfused with RPMI culture media containing 10U/mL heparin for 5 min (1 ml/min flow rate) before the brains were removed. Brain lysates were generated by homogenizing whole brains with a mechanical tissue homogenizer (Arrow engineering CO), followed by sonication (3 cycles of 3 s each at amplitude 30 %; Sonifier 450 (Branson Ultrasonics, Brookfield, Connecticut) in 3 mL/g of Lysis Buffer (20 mM Tris,150 mM NaCl, 0.5 % Tween-20, 1X cOmplete Mini tablets EDTA-free [Millipore Sigma. St Louis, MO]). Lysates were cleared by centrifugation at 9300 x g for 15 min at 4 °C. Levels of 18 cytokines and chemokines were measured from 50 μL of plasma and brain lysates (adjusted to 10 mg/mL) via a Luminex multiplex panel (Mouse High Sensitivity 18-Plex Discovery Assay). Measurements performed by Eve Technologies (Calgary, Canada).

### 2.8. Flow cytometry

Flow cytometry was performed similarly to our earlier work, but with some modifications (Velloso et al., 2022a). In short, following perfusion with RPMI culture media containing 10U/mL heparin for 5 min (1 ml/min flow rate), whole brain tissue from mice at P7 was digested with 0.1 U/mL of Liberase-DH (Millipore Sigma. St Louis, MO) for 15 min. followed by gentle mechanical trituration. The cells were incubated on ice for 20 min with the antibodies listed in Table 1. Dead cells were excluded from staining with Zombie UV (Biolegend, San Diego, CA). Isotype controls of the same fluorophore and isotypes as the antibodies in Table 1 were used to determine non-specific staining. Flow cytometry was performed on an LSRFortessa X-20 and analyzed using FlowJo 10.9 (BD Biosciences, Franklin Lakes, NJ).

### 2.9. Immunofluorescence

Mice were anesthetized with 350 mg/kg Avertin at P7 and transcardially perfused with RPMI culture media containing 10U/mL heparin for 5 min, followed by perfusion with 3 % paraformaldehyde. After cryoprotection in 30 % sucrose, the brains were sectioned sagittally at 20 μm and stained for either GFAP (GF2.2 mAb, 1:1) to assess astrocytes or with Iba1 (Wako chemicals 019-19741, 1:300) and CD68 (Bio-Rad MCA1957GA, 1:200) to evaluate changes in microglia. Cell numbers and morphometric parameters were quantified using CellProfiler (Stirling et al., 2021) by averaging four 20X frontal cortex images per animal and three animals per experimental group, all from different litters.

### 2.10. RT-qPCR

Mice were anesthetized with Avertin at P7 and underwent *trans*-cardiac perfusion with RPMI culture media containing 10U/mL heparin for 5 min to remove blood cells. The brain was removed, and the frontal cortex was isolated by cutting the brain in the coronal plane at Bregma 2.0 mm (rostral to the caudate-putamen) and removing the anterior olfactory area. The tissue was frozen in Tri-Reagent (Sigma-Aldrich, T9424, Saint Louis, MO). mRNA was extracted using the RNeasy Mini Kit (Qiagen, 74104, Hilden, Germany. mRNA was converted to cDNA using iScript (Bio-Rad, cat #1708890, Hercules, California,) and qPCR was performed using SYBRgreen (Bio-Rad, Cat #1725121) with the touchdown method using 100 ng of RNA per replicate (Zhang et al., 2015). Primers for the following genes were used: IL-1a, IL-4, IL-6, IL-10, IL-13, IL-17a, and Leukemia Inhibitory Factor (LIF) (Table 2). Gene expression was normalized to Ppia (Peptidylprolyl Isomerase A). Gene expression was analyzed with a 2-factor ANOVA for sex and treatment conditions.

### 2.11. Statistical analyses

For *in vivo* studies, at least eight animals, from at least 3 litters were examined per group at each time point. The analysts were blinded to the experimental condition for all experiments where data points were scored manually. Statistical analyses were performed using GraphPad Prism software V10.4 (San Diego, CA). Sample size tests (power analysis) were performed to estimate the group sizes required for each experiment based on pilot studies (G*Power; University of Dusseldorf). Graphs are presented as violin plots to express the complexity of the data. For experiments with continuous variables, results were analyzed for statistical significance using a two-tailed, unpaired Student’s *t*-test. The means between more than two groups were compared using one-way ANOVA or two-way ANOVA whenever more than one variable was present. Bonferroni’s correction was used for multiple comparisons. Comparisons were interpreted as significant when p < 0.05. Normality (gaussian) tests (Kolmogorov-Smirnov) were performed before tests that assumed normal distributions. Sphericity corrections were performed for ANOVA tests. Outliers were identified and removed from analyses using Prism’s ROUT method (Q = 1 %).

## 3. Results

We previously showed that transiently increasing systemic levels of IL-6 by around 2-fold in neonatal mice affects communication, sociability, and repetitive behaviors as early as postnatal day 42 (P42) (Velloso et al., 2022b). In that previous study, the effects of neonatally elevated IL-6 on behavior remained evident as late as P120, indicating that early changes induced by IL-6 were sustained late into adulthood. However, it was not clear how early behavior changes could be observed after the IL-6 treatment regimen. Secondly, although our data indicated that IL-6 directly impacted neural cell development (Kumari et al., 2020), the sustained effects of IL-6 administration could result from secondary neuroinflammation. We postulated that if IL-6 directly affects neurodevelopment, its impact on the three core behavioral domains (communication, sociability, and repetitive behaviors) should be observed acutely after elevating IL-6. To test his hypothesis, we treated mice systemically with IL-6 following the same injection paradigm as previously employed (Velloso et al., 2022b) and then evaluated the three core behavioral domains using age-appropriate tests between P7 and P21.

### 3.1. IL-6-treated mice have altered patterns of ultrasonic vocalizations

**Fig. 1.**
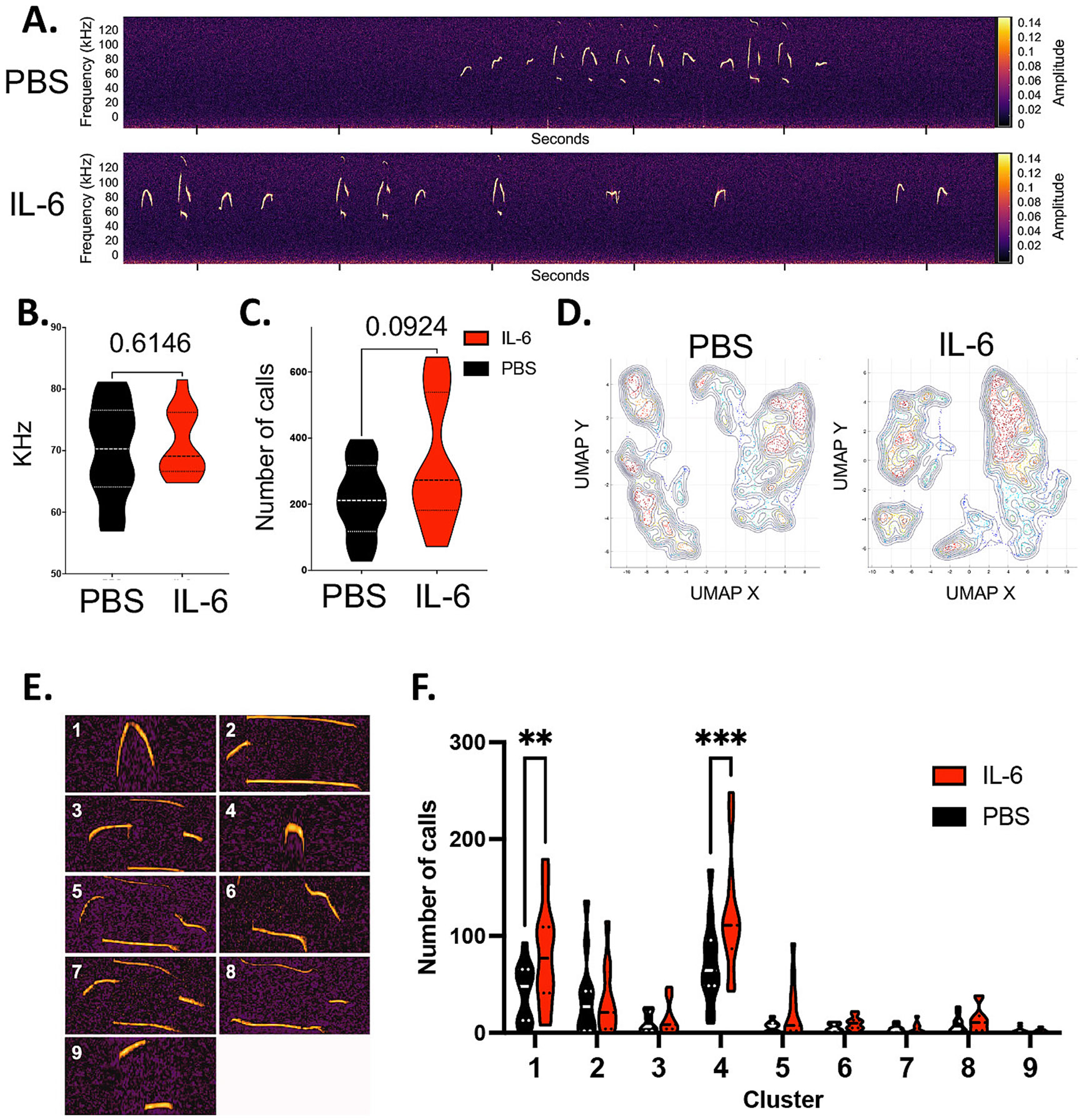
IL-6-treated male mice have altered patterns of ultrasonic vocalizations. (A) Representative sonograms of ultrasonic vocalizations produced during nest isolation by P7 male mice injected with either PBS or rmIL-6. Sonograms were analyzed using DeepSqueak. (B) Principal frequency of calls produced during nest isolation, p = 0.615 by unpaired *t*-test, t = 0.5108, df = 22, F = 2, n = 24, outliers = 0. (C) Total number of ultrasonic calls, p = 0.0924 by unpaired *t*-test, t = 0.1.759, df = 22, F = 3.024, n = 24, outliers = 0. (D) Unsupervised UMAP clustering using combined vocalizations from all PBS and IL-6 treated mice. (E) Representative vocalizations for 9 clusters created by supervised clustering based on call morphologies. (F) Number of vocalizations by cluster in the 9 clusters represented in E. ** p < 0.01, *** P < 0.001 by two-way ANOVA multiple comparisons, t = 3.441(1) and 4.229(4), df = 187, n = 24, outliers = 0.

**Fig. 2.**
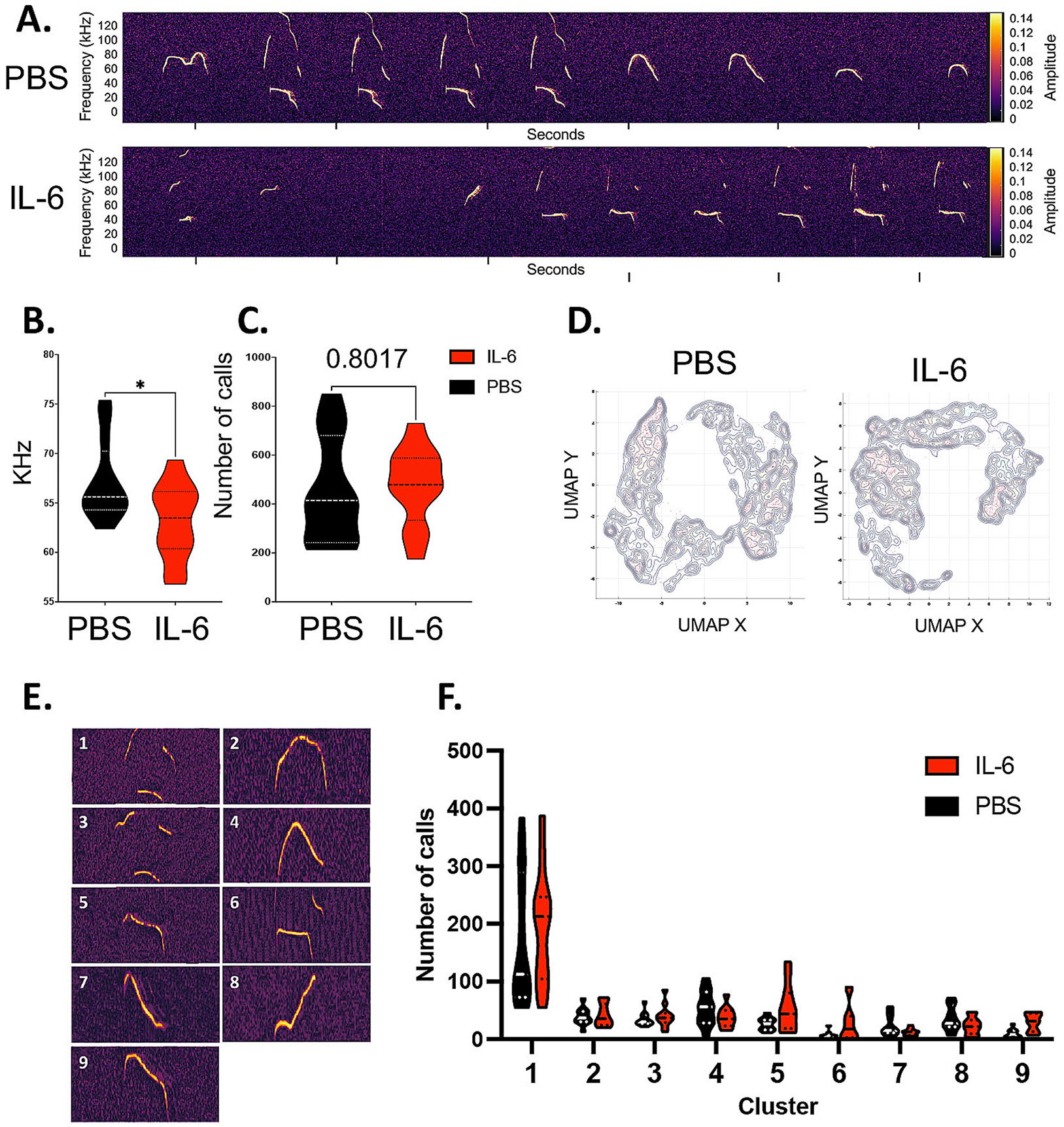
IL-6-treated female mice have altered patterns of ultrasonic vocalizations. (A) Representative sonograms of ultrasonic vocalizations produced during nest isolation by P7 female mice injected with either PBS or rmIL-6. Sonograms were analyzed using DeepSqueak. (B) Principal frequency of ultrasonic calls produced during nest isolation, * p < 0.05 by unpaired *t*-test, t = 2.48, df = 22, F = 1.142, n = 24, outliers = 0. (C) Total number of ultrasonic calls. p = 0.8017 by unpaired student *t*-test, t = 0.2543, df = 22, F = 1.1824, n = 24, outliers = 0 (D) Unsupervised UMAP clustering using combined vocalizations from all PBS and IL-6 treated mice. (E) Representative vocalizations for 9 clusters created by supervised clustering based on call morphologies. (F) Number of vocalizations by cluster in the 9 clusters represented in E.

We started by evaluating ultrasonic vocalizations (USV), which mice produce across different social contexts to communicate with each other (reviewed in (Caruso et al., 2022). USVs have been extensively used for early and adult behavioral phenotyping of rodent models of neurodevelopmental disorders, showing quantitative (number of ultrasonic calls) and qualitative (morphology of vocal repertoire) changes (Caruso et al., 2020). During neonatal development, mouse pups emit USVs when isolated from the nest. These isolation-induced USVs elicit maternal orientation/approach and peak in rate during the first postnatal week (Elwood and Keeling, 1982). We recorded USVs from male and female mouse pups individually isolated from the nest at P7 immediately following the injections performed between P3 and P6. Quantitative analyses of the physical characteristics of the USVs (number of calls, frequency, power, amplitude duration) performed in SASlab (data not shown) and DeepSqueak (Coffey et al., 2019) for the male mice (Fig. 1A) showed no change in physical characteristics (Fig. 1B) but revealed a trend (p < 0.1 by unpaired student *t*-test, n = 12 per group) in the IL-6 injected pups (henceforth IL-6 Rx) for producing a higher total number of calls during the 5 min isolation period than the PBS injected littermate controls (Henceforth PBS Rx) (Fig. 1C). To investigate the composition of the vocal repertoire of these mice, we performed qualitative spectrographic analysis, clustering the calls by morphology. Unsupervised UMAP clustering revealed changes in the size and distribution of the call populations produced by the IL-6 injected mice compared to their control littermates (Fig. 1D). A second unsupervised clustering combining IL-6 and PBS samples classified nine types of distinct vocalizations (Fig. 1E). These nine cluster identities were used to train a classification neural network that was then used to perform supervised clustering on PBS and IL-6 Rx mouse vocalizations (Fig. 1E,F). Comparing the clusters revealed that the number of calls produced by the IL-6 Rx male mice was significantly higher in the clusters with simpler morphologies (clusters 1 and 4, * p < 0.05, ** P < 0.01, n = 12, by unpaired student *t*-test). Clusters with more complex morphologies were similar between IL and 6 Rx and PBS Rx mice (Fig. 1E,F). By contrast, this analysis showed that the female mice produced a similar number of calls (Fig. 2C), with no changes in morphology (Fig. 2E,F), but instead at a lower frequency (Fig. 2B). These experiments indicate that IL-6 Rx produces sex-specific qualitative changes in communication patterns that can be detected immediately after administration.

### 3.2. Both male and female mice exposed to IL-6 postnatally are retrieved less often after nest isolation

**Fig. 3.**
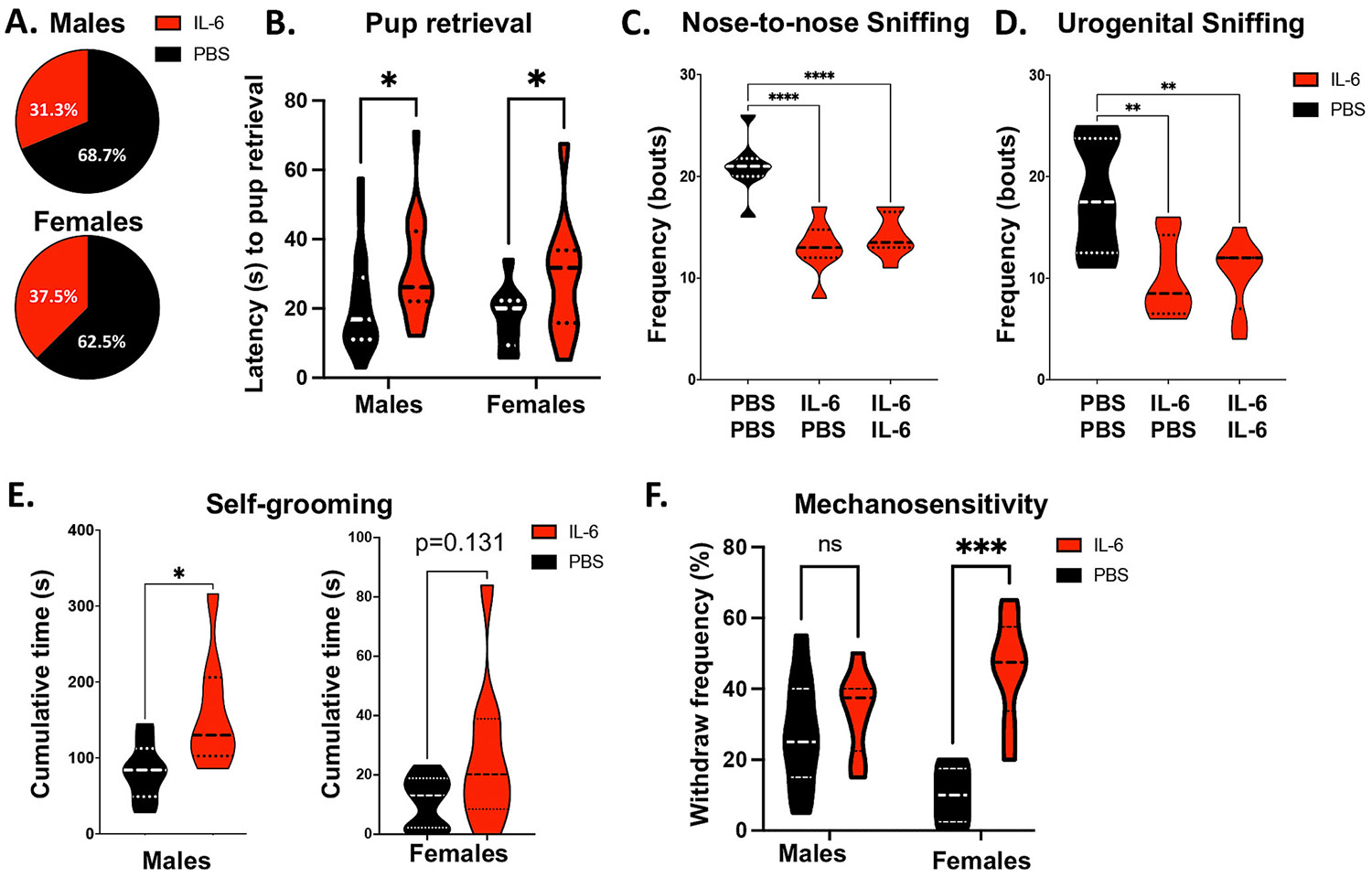
Mice exposed to IL-6 postnatally are retrieved less often after nest isolation, are less sociable and have increased repetitive behaviors and mechanosensitivity as juveniles. Pairs of sex-matched P7 IL-6 and PBS-treated mice were removed from the nest and placed on opposite sides of the home cage, while the mother was placed in the nest and allowed to retrieve pups back to the nest. The frequency of maternal first choice for pup retrieval (A) and the total latency for pup retrieval back to the nest (B) were recorded. * P < 0.05 by two-way ANOVA multiple comparisons, t = 2.713, df = 55, n = 63, outliers = 2 PBS/3 IL-6. Frequency of nose-to-nose sniffing (C) and urogenital sniffing (D) behaviors for IL-6 Rx mice were assessed in pairs of control mice (PBS/PBS), test mice (IL-6/IL-6) and mixed pairs (PBS/IL-6) during the 5-minutes of social play. ** P < 0.01, **** p < 0.0001 by one-way ANOVA multiple comparisons, t = 6.288 (in C) and 3.622 (in D), df = 21, n = 24, outliers = 0. (E) Cumulative duration of self-grooming measured in a clean, isolated environment over 5 min for males and females. * p < 0.05, by unpaired student *t*-test, t = 2.562, df = 14, F = 3.976, n = 16, outliers = 0. (F) Mechanical sensitivity was evaluated in IL-6 and PBS-treated male and female mice at P13 using a paradigm that measures the frequency of hind-paw withdraw elicited by a 0.4 g resistance Von Frey filament. *** P < 0.001 by two-way ANOVA, t = 1.283, df = 36, n = 40, outliers = 0.

Nursing mouse mothers promptly retrieve pups that exit the nest in response to pup vocalizations. To evaluate whether impaired pup vocalizations would affect this maternal behavior, we evaluated pup retrieval delay in IL-6 Rx and PBS Rx pup dyads. IL-6 Rx males and female pups were more frequently retrieved last (Fig. 3A), and it took longer for the dam to return them to the nest compared to control littermates (Fig. 3B). Following a report that the vocalizations that prompted pup retrieval depended on specific bursts of calls (Valtcheva et al., 2023), we evaluated the occurrence of USV bursts. However, there were no differences in male or female IL-6 Rx from controls after binning the vocalization intervals to find bursts of short-interval calls.

### 3.3. IL-6-treated male mice are less social as juveniles and have increased repetitive grooming behavior

Juvenile mice naturally play with their peers, and their play is evident in many distinct behaviors (e.g., sniffing, following, and pushing/crawling) that can be classified and quantified to assess sociability (McFarlane et al., 2008; Silverman et al., 2010). These behaviors are usually abnormal in male mouse models for ASD (Dawson et al., 2023; Zarate-Lopez et al., 2024). To evaluate sociability in the IL-6 Rx male mice, we scored behaviors during reciprocal social interactions (juvenile play) at P21. Naïve mouse dyads were allowed to interact for 10 min in a neutral environment, and social behaviors (sniffing, following, pushing/crawling) were manually scored by an operator blinded to the treatment group. We paired IL-6 Rx mice in dyads with either a control or another IL-6 Rx littermate to test whether social engagement prompted by a control partner would stimulate a response in the IL-6 Rx mice. We did not observe differences in pushing/crawling or following behaviors (data not shown); however, when paired with PBS Rx or other IL-6 Rx partners, the mice that had received IL-6 neonatally engaged far less frequently in both nose-to-nose (Fig. 3C) and urogenital (Fig. 3D) sniffing (**** P < 0.0001, ** P < 0.01, unpaired Student’s *t*-test, n = 8 per group,). These two sniffing behaviors are the main hallmarks of social engagement in young mice, indicating that a transient increase in circulating IL-6 during development results in a decrease in social behaviors that can be detected as early as P21.

### 3.4. Male mice treated with IL-6 postnatally have increased self-grooming behavior

Exacerbated self-grooming behavior in mice is analogous to the increased repetitive or stereotyped behaviors that are diagnostic criteria for ASD (American Psychiatric Association, 2013) that are often observed in other neurodevelopmental disorders (Whitehouse and Lewis, 2015). We previously found that self-grooming was exacerbated in P56 IL-6 Rx male and female mice. Therefore, we evaluated grooming behavior at P21 for male and female mice. This analysis demonstrated that, like the older mice, juvenile IL-6 Rx male mice spent significantly more time engaging in self-grooming behavior when compared to PBS Rx mice (Fig. 3E) (P < 0.05, unpaired Student’s *t*-test, n = 8). By contrast, P21 female mice had only a trend of increased self-grooming behavior (Fig. 3E), indicating that the emergence of this phenotype is delayed in the females.

### 3.5. Female mice treated with IL-6 postnatally have increased mechanosensitivity

In addition to the three core behavioral features of ASD, approximately 95 % of individuals on the spectrum have sensory abnormalities, with ~ 60 % displaying altered tactile sensitivity (Tomchek and Dunn, 2007), with hypersensitivity being the most commonly reported sensitivity associated with ASD (Puts et al., 2014). Tactile sensitivity alterations are also commonly observed in children with attention-deficit/ hyperactivity disorder (ADHD), presenting with disorder-specific characteristics when compared to ASD (He et al., 2021).

We evaluated mechanical sensitivity in the IL-6 and PBS Rx mice at P13, by measuring the frequency of hindpaw withdraws elicited by 0.4 g resistance Von Frey filaments. This analysis showed that the IL-6 Rx mice had increased tactile sensitivity compared to the PBS Rx mice (P < 0.001, Two-way ANOVA, F (1,28) = 17.86). However, to our surprise, only the female IL-6 Rx mice exhibited this hypersensitivity phenotype (P < 0.001 by two-way ANOVA post-hoc, n = 13 per group), while male mice showed no difference in mechanosensitivity when compared to sex-matched PBS Rx controls (Fig. 3F). Importantly, although there was a trend for the female mice to have baseline reduced tactile sensitivity, male and female PBS Rx groups were not significantly different, while IL-6 Rx females were more sensitive that the PBS Rx males (P = 0.12 and P < 0.05, respectively, by two-way ANOVA post-hoc, n = 13 per group). These data indicate that IL-6 elicits a strong tactile hypersensitivity phenotype exclusively in the females at P13.

### 3.6. Transiently elevating systemic IL-6 leads to a sustained mild increase in core body temperature, with no impact on growth

**Fig. 4.**
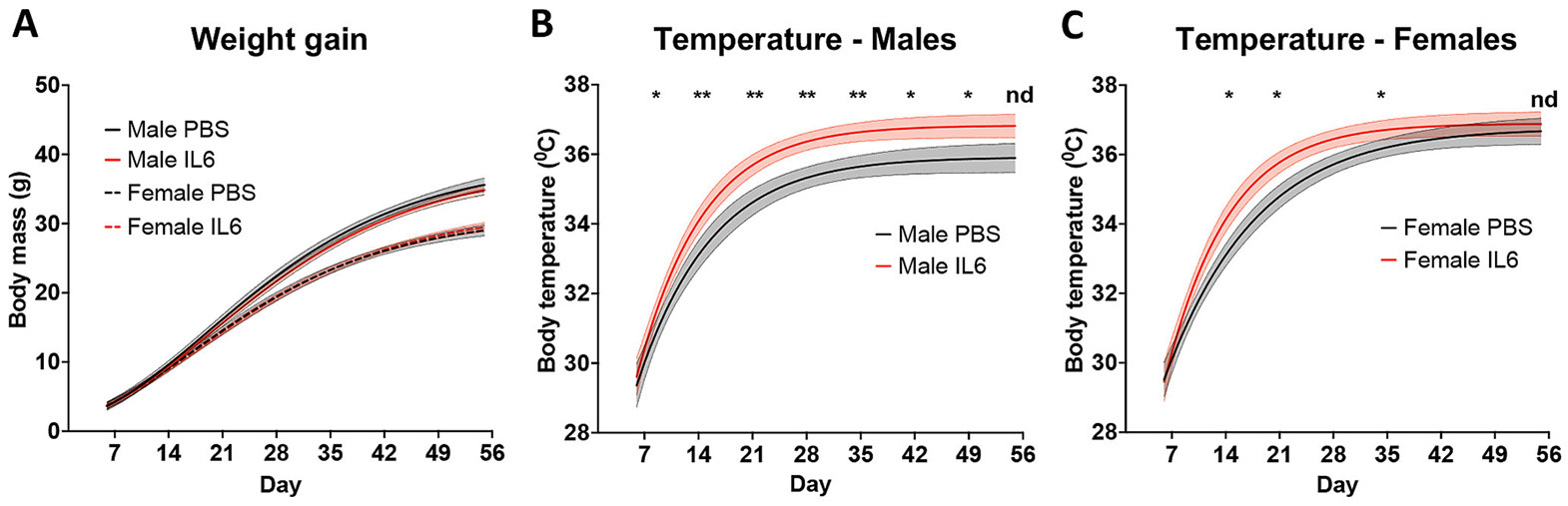
Modestly elevating systemic IL-6 for 3.5 days postnatally increases body temperature. Body mass (A) and temperature (B-C) were tracked after IL-6 treatment for male and female mice until P56. Body temperature and weight were measured every two days between P7 and P14 and weekly thereafter. Lines represent nonlinear regression with 95 % confidence bands. * p < 0.05, ** P < 0.01 by two-way ANOVA multiple comparisons. Effect of IL-6 treatment by ANOVA. Males: ** p < 0.0001, F(1,13) = 29.89, n = 15, outliers = 0. Females: * p = 0.0139, F(1,12) = 8.285, n = 14, outliers = 0.

Since the behavioral assessments showed robust effects of the IL-6 Rx regimen on social, communication and repetitive behaviors, we were interested in whether they were due to sustained systemic inflammation. Our past data indicated that systemically-injected IL-6 directly affected neural stem cells and progenitors of the subventricular zone (Kumari et al., 2020); however, the mediating effect of other cytokines or immune cells was not explored. Therefore, to address this question, we compared the core body temperature of male and female IL-6 Rx mice to littermate controls as a readout for elevated levels of pro-inflammatory cytokines known to be pyrogenic. We measured body temperature every two days, beginning at P7 until P14, and then weekly thereafter until P56. IL-6 Rx males had a slight (~1.5C) but consistent increase in body temperature until P49 (Fig. 4B, * p < 0.05, ** P < 0.01 by two-way ANOVA. n = 16 per group). Females, however, showed a milder response to the IL-6 Rx, showing increased temperatures at P14, P21, and P35 (33.9 vs 34.5, 34.4 vs 36, 36.3 vs 37, respectively) (Fig. 4C, * p < 0.05, by two-way ANOVA. n = 16 per group). Surprisingly, the IL-6 Rx did not affect the body weights of either male or female cohorts during this same period (Fig. 4A).

### 3.7. IL-6-treated mice have acute changes in circulating cytokines

**Fig. 5.**
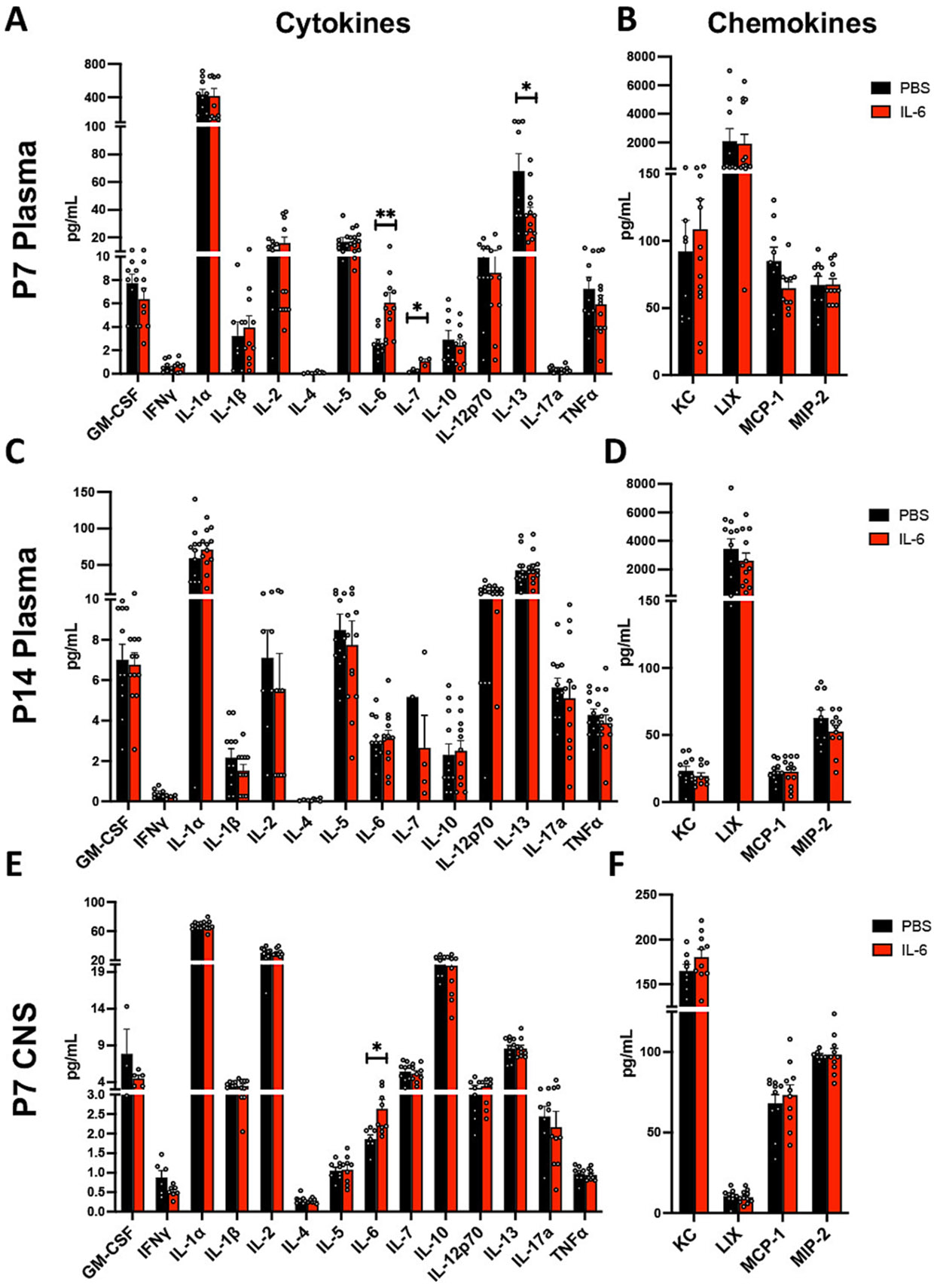
Mice exposed to IL-6 postnatally have acute changes in circulating and CNS cytokine and chemokine levels. Acute circulating cytokine (A) and chemokine (B) levels (pg/mL) measured at P7 in the plasma of PBS and IL-6-treated male and female mice. * p < 0.05, ** p < 0.01 by unpaired student *t*-test. IL-6: t = 3.205, df = 19, F = 7.764, n = 21, outliers = 1 IL-6. IL-7: t = 3.923, df = 4, F = 5.526, n = 6, outliers = 0. IL-13: t = 2.577, df = 20, F = 4.44, n = 22, outliers = 0. No sustained changes at P14 in circulating cytokine (C) and chemokine (D) levels (pg/mL) measured in the plasma of PBS and IL-6 male and female treated mice. n = 12 per group. Acute cytokine (E) and chemokine (F) levels (pg/mL) measured at P7 in brain lysates of male and female mice that received intraperitoneal injections of either PBS or IL-6. * p < 0.05, by unpaired student *t*-test, t = 2.371, df = 15, F = 6.495, n = 17, outliers = 2 PBS/1 IL-6.

The increase in body temperature in the IL-6 Rx mice could reflect systemic inflammation, where an acute spike in IL-6 could stimulate circulating immune cells to secrete other cytokines that would then be self-perpetuating. Therefore, we evaluated how the IL-6 Rx affected the levels of 14 circulating cytokines (GM-CSF, IFNγ, IL-1α, IL-1β, IL-2, IL-4, IL-5, IL-6, IL-7, IL-10, IL12p70, IL-13, IL-17a, TNFα) and 4 chemokines (KC [CXCL1], LIX [CXCL5], MCP-1 [CCL2] and MIP-2 [CXCL2]) using a Luminex 18-plex panel. As expected, the level of plasma IL-6 in the IL-6 Rx mice was increased by ~ 2.5-fold when measured immediately after the end of the IL-6 Rx (P7) (Fig. 5A, ** p < 0.01 by unpaired student *t*-test. n = 13 per group), which was more pronounced in the males (Fig. S1) and consistent with our previous measurements made using by ELISA (Kumari et al., 2020). Interestingly, the only other acute effects resulting from this increase in IL-6 were a 4-fold increase in IL-7 and a ~ 45 % reduction in circulating IL-13 (Fig. 5A,B, * p < 0.05 by unpaired student *t*-test. n = 13 per group) with no sex differences (Fig. S1). Contrary to our predictions, these changes in cytokine levels were not sustained at P14, when no differences in plasma cytokine or chemokine levels were detected in IL-6 Rx mice compared to PBS Rx controls (Fig. 5C,D). To our surprise, the levels of IL-6 were also normalized, indicating that the excess IL-6 had already been cleared 7 days post-treatment. These data strongly suggest that the increased body temperature seen in the IL-6 Rx mice is not the result of widespread systemic inflammation and that, in fact, mildly elevating circulating IL-6 in neonates does not elicit the production of other pro-inflammatory cytokines. Moreover, the acute spike in circulating IL-6, from P7 to P14, indicates that this cytokine acts during a short window to produce the behavior deficits we reported here and in previous studies.

### 3.8. Mice treated with IL-6 systemically have an acute increase in CNS levels of IL-6

Although published studies have shown that IL-6 is transported across the Blood Brain Barrier (BBB) via saturable transporters, (Banks et al., 1995; Banks et al., 1994) it was necessary to confirm that the systemically delivered IL-6 entered the CNS. Moreover, we wanted to establish whether increased levels of other cytokines or chemokines might impact neural development. Therefore, we measured acute CNS levels of IL-6 and 17 other cytokines and chemokines in IL-6 and PBS Rx whole brain lysates. At P7, the only measurable difference in cytokine or chemokine CNS levels was a 40 % increase in IL-6 in the IL-6 Rx mice, compared to controls (Fig. 5E,F; * p < 0.05, by unpaired student *t*-test. n = 9 per group), with no sex differences (Fig. S1).

To establish whether the increase in IL-6 might not be due to transport across the Blood-Brain-Barrier but was rather due to production by resident brain cells, we extracted mRNA from the frontal cortex of IL-6 Rx and control mice and found that the expression of IL-6 was unchanged (Table 3). We also evaluated levels of IL-1α, IL-4, LIF, IL-10, IL-13, and IL-17a in the frontal cortex. IL-13 expression was significantly higher in females than males with no effect of treatment (Student’s *t*-test, t = 7.83, p = 0.016, n = 14 mice per group, outliers = 1). Levels of IL-1α, IL-4, and LIF were undetectable in most samples and, therefore, not analyzed. IL-6, IL-10, and IL-17a had equal expression between sexes and treatment conditions, and no cytokines had significant interactions between treatment condition and sex (Table 3).

## 4. 3.9: Mice exposed to IL-6 postnatally do not show changes in infiltrating or resident immune cells in the brain

**Fig. 6.**
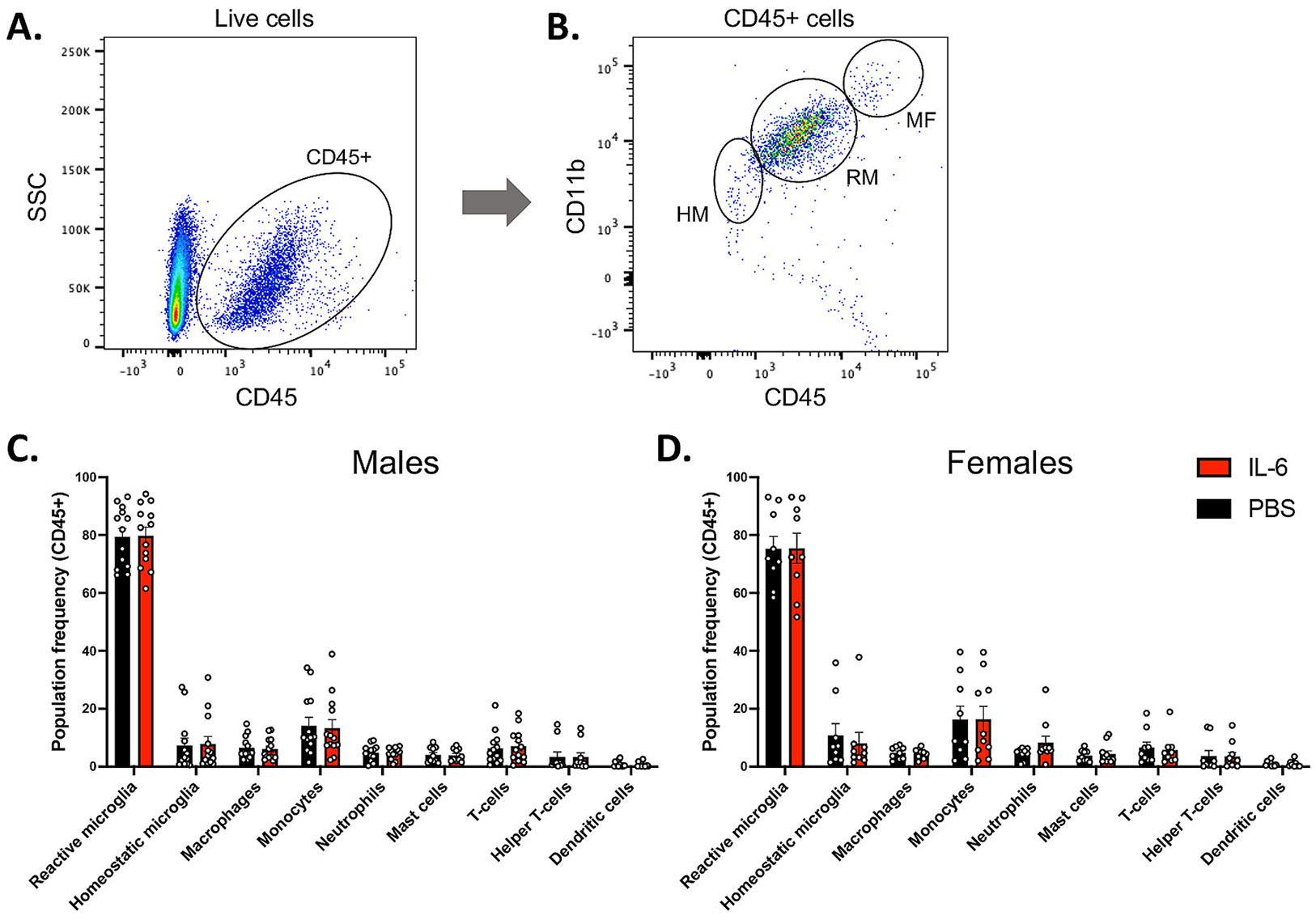
Mice exposed to IL-6 postnatally do not show changes in infiltrating or resident immune cells in the brain. Flow cytometry identification of immune cells in whole brains of PBS and IL-6 injected P7 male and female mice. (A) CD45 + cells were gated from the population of live cells. (B) CD11b and CD45 plots were used to gate Homeostatic Microglia (HM), Reactive Microglia (AM) and Macrophages (MF). Other immune related cell types were similarly gated from the population of CD45 + cells using CD11b, Ly-6G, Ly6C, CD3, CD4, CD117 and F4/80 and are expressed as frequency in the CD45 + population for male (C) and female (D) mice. n = 24 per group.

Having established that systemic IL-6 Rx increased the levels of IL-6 in the brain without affecting other cytokines, it was important to determine whether the increased IL-6 stimulated the infiltration of peripheral immune cells into the CNS and/or the activation of the brain’s resident immune cells (Lacroix et al., 2002). Therefore, we evaluated the immune cell composition in the IL-6 Rx mice using flow cytometry. We gated on the CD45 + cells and further discriminated other cell types using a 9-color flow cytometry panel (Fig. 6A,B). At P7, there were no differences in the numbers of any subsets of immune cells in the IL-6 Rx vs the PBS Rx controls (Fig. 6C,D). Numbers of macrophages, monocytes, neutrophils, mast cells, T-cells, and dendritic cells were unchanged in the IL-6 injected mice (Fig. 6C,D). Again, contrary to our expectations, the proportion of homeostatic (CD45^low^/CD11b^+^) to reactive microglia (CD45^Int^/CD11b^+^) was unchanged in IL-6 Rx mice, indicating that the resident immune cells were not activated by the increase in CNS IL-6 levels (Fig. 6C,D).

To further investigate microglia states, we evaluated the microglia in the frontal cortex acutely after IL-6 injections. Immunohistochemical analysis revealed no change in the number of Iba1 + microglia, Iba1/CD68 double positive, reactive microglia or the number of CD68 speckles per Iba1 + microglial cell (Fig. S2). We also measured the branching complexity (number and length of branches per cell) of the Iba1 + cells and found no effect of IL-6 Rx, for both male and female mice (Fig. S2). We also evaluated astroglial responses by staining for GFAP and did not see any change in GFAP intensity (Fig. S3). Males and females had very similar numbers of immune cells by flow cytometry suggesting that there were no underlying sex-specific differences in response to the IL-6 Rx (Fig. 6C,D; Fig. S1, S2).

## 5. Discussion

Immune activation models have consistently shown that pups that were exposed to either maternal inflammation in utero or pups that have been exposed to cytokines immediately after birth have aberrant communication, sociability, and repetitive behaviors as adults. These signals represent the core behavioral domains in the diagnostic criteria for ASD (American Psychiatric Association, 2013). However, in humans, signs of ASD can be reliably detected in two-year-olds, a developmental age that correlates to mice right after weaning. Thus, here we investigated whether aberrant social, communication, and repetitive behaviors could be detected in pre-pubescent juvenile mice.

We had previously shown that IL-6 alters the proliferation and subsequent fates of stem cells and progenitors from the secondary germinal matrices of the brain, specifically the subventricular zone (SVZ) and subgranular zone (SGZ) (Kumari et al., 2020). By employing Nestin-Cre reporter mice to track the descendants of the neural stem cells, we showed that neonatal IL-6 administration reduced the production of astrocytes in the amygdala and frontal cortex and reduced neurogenesis in the dentate gyrus of the hippocampus (Velloso et al., 2022b). We concluded that a short exposure to IL-6 altered the trajectory of neural development, resulting in abnormal synaptic connectivity and behavioral changes. However, we could not exclude the possibility that neuroinflammation, immune cell infiltration or elevated secondary cytokines could orchestrate these changes, leading to altered behaviors. Therefore, here we investigated the presence and activation of resident and infiltrating immune cells and levels of CNS and circulating cytokines and chemokines acutely after the initial IL-6 administration and then one week later to establish whether the IL-6 induced prolonged neuroinflammation.

Low levels of IL-6 are present in the CNS under normal physiological conditions throughout development (Rothaug et al., 2016) and the expression of IL-6 is tightly controlled in the developing human and mouse brain (Gruol, 2015). Elevated levels of IL-6 are associated with several neurodevelopmental disorders, including Attention Deficit Hyperactivity Disorder (Misiak et al., 2022), Tourette syndrome (Li et al., 2022), Schizophrenia (Patel et al., 2022) and Autism Spectrum Disorder (ASD) (Rudolph et al., 2018; Smith et al., 2007; Spann et al., 2018). Animal models for ASD that aim to mimic gestational inflammation have consistently shown elevated levels of IL-6 that correlate with abnormal behaviors in the adult offspring. We showed that IL-6 is sufficient to cause behavioral abnormalities, as injecting IL-6 into neonates produced a range of functional changes that were sustained into late adulthood (Velloso et al., 2022b). In some Maternal Immune Activation (MIA) models, IL-6 levels remain elevated long after the initial perturbation (Samuelsson et al., 2006; Samuelsson et al., 2004), mirroring epidemiological data that show that children with autism have high serum levels of IL-6 that persist until at least 4 years of age (Ashwood et al., 2011). This raised the question as to whether injecting IL-6 into neonatal mice would create a feed-forward cycle to maintain increased levels in the CNS or whether it would promote the persistent production of other cytokines. Within the inflamed CNS, IL-6 can be secreted by immune cells (T-cells, B-cells, macrophages and microglia) (Rose-John et al., 2007) and astrocytes in response to a variety of signals, including of IL-6 itself (Choi et al., 2014; Marz et al., 1999; Sofroniew, 2014). Isolated microglia readily respond to IL-6 as they strongly express the IL-6 receptor (Lin and Levison, 2009), along with leukocytes (Oberg et al., 2006) and some restricted progenitors (Kumari et al., 2020). IL-6 stimulates microglia to secrete other cytokines and chemokines that include CCL1, CXCL1, MIP-1α/β, IL-8, IL-13, IL-16, IL-18, MIF and Serpin-E1 (Couch et al., 2023). Therefore, it was conceivable that an acute increase in IL-6 would create a cascade effect, maintaining elevated CNS levels of IL-6 and possibly other proinflammatory cytokines. Contrary to this prediction, we observed that elevating systemic IL-6 in young mice did not induce acute production of other cytokines or chemokines in either the circulatory or nervous systems, other than a modest increase in serum IL-7, which was present at very low levels in only 23 % of our samples, and, therefore, does not likely have any physiological impact. We also did not observe any sex differences in circulating or CNS cytokines levels. Moreover, all cytokines, including IL-6, returned to baseline within a week’s time, indicating that the administered IL-6 dose did not trigger a forward feedback pro-inflammatory cascade.

An interesting finding was the nearly 50 % reduction in serum IL-13. Although usually associated with eosinophilic asthma (Matsuyama et al., 2024), this cytokine is required for cortical inhibitory synapse maturation (Barron et al., 2023). In this recent study, Barron et al. showed that IL-13 is produced by innate lymphoid cells from P5 to P15, the same period when we observed a reduction in this cytokine. Loss of the IL-13-producing cells or deletion of the IL-13 receptor in inhibitory neurons was sufficient to decrease cortical inhibitory synapses and produced impairments in adult social behavior (Barron et al., 2023).

Our findings are in contrast with some studies that investigated cytokine levels in MIA mouse models. In several studies, mid-gestational administration of the viral mimetic Poly I:C produced high levels of the proinflammatory cytokines IL-1β, IL-6, IL-10, and TNF-α in the maternal circulation, placenta and brain (Desbonnet et al., 2022; Garay et al., 2013; Hsiao and Patterson, 2011; Meyer et al., 2006; Smith et al., 2007; Zhang et al., 2021). Increased levels of IL-1β, IL-10, IL-6 and TNF-α were subsequently found in the fetal brains (Hodyl et al., 2007; Meyer et al., 2006) and in the circulation of the resulting young (Chamera et al., 2020) and adult offspring (Mueller et al., 2021). Conversely, other studies found that increased levels of IL-6, IL-17, and IFN-γ were no longer detected in the offspring of Poly I:C injected mice at 3 weeks of age (Carbone et al., 2023; Majerczyk et al., 2022; Shimizu et al., 2021). This variability was highlighted by Mueller et al., 2021, who showed that the progeny of a large cohort of isogenic C57BL6/N mice exposed to identical MIA conditions could be stratified into distinct subgroups based on behavior phenotype and cytokine expression. Specifically, only a subset of mice displayed elevated peripheral IL-1β, IL-6, and TNF-α into adulthood (Mueller et al., 2021). Despite the variability in cytokine profiles generated by different MIA strategies, it is essential to point out that IL-6 is consistently a hub cytokine linking immune challenges to behavioral phenotypes. Notably, injecting IL-6 alone at either mid- or late gestation into pregnant rats leads to reduced spatial learning and sustained high levels of circulating IL-6 in the offspring up until 24 weeks (Samuelsson et al., 2006; Smith et al., 2007). We selected a postnatal dose that led to a two-fold increase in circulating IL-6, which might explain the limited downstream effects on other cytokines. To further demonstrate the crucial role of IL-6, administering anti-IL-6 antibodies is sufficient to prevent the behavioral phenotypes produced by mid-gestational administration of Poly I:C in two separate studies (Lipina et al., 2013; Smith et al., 2007).

The more pronounced effects of gestational inflammatory insults compared to postnatal strategies may be due to the evolving permeability of the placental and blood–brain barrier (BBB) at different developmental stages. IL-6 crosses the BBB via a controlled saturable transport (Threlkeld et al., 2010); however, only a small fraction of the plasma IL-6 crosses the BBB. One study that used radiolabeled IL-6 found approximately 0.2 % of the dose injected i.v. into adult mice could be detected per gram of brain tissue (Banks et al., 1994). Surprisingly, the recovery of ^125^I-IL-6 injected dams at mid-gestation was 40-fold higher in fetal tissue, suggesting that the permeability to IL-6 of the placental and BBB markedly changes during development (Dahlgren et al., 2006). This is in accordance with a report that microglia morphological changes are only observed in fetuses when Poly I:C is injected to dams at mid- but not late gestation (Ozaki et al., 2020). In our experiments, acute levels of IL-6 were increased 2-fold in the circulation 24 h after injections, but only a fraction of this IL-6 reached the brain, represented by the 40 % increase of IL-6 in the CNS at the same time point. Increased CNS levels of IL-6 likely represent BBB transport, as local mRNA expression was not elevated. Future studies using labeled IL-6 will corroborate the exogenous origin of the elevated CNS IL-6.

The moderate levels of IL-6 that reached the CNS in our model do not appear to promote neuroinflammation, as we did not observe increased mRNA expression or increased protein level for any other cytokine or chemokine. A limitation of these analyses was that the cytokines and chemokines were measured in whole brain, possibly masking regional changes. As levels of IL-6 normalized in the serum by seven days after the IL-6 increase, IL-6 must be exerting its effects on neural development within the first week of murine life: a period when synaptogenesis is peaking (Dutta and Sengupta, 2016; Semple et al., 2013) and SVZ and SGZ progenitors are rapidly expanding. Collectively, these data support our initial hypothesis that IL-6 has direct effects on neural progenitors and their maturation rather than triggering neuroinflammation (Kumari et al., 2020; Velloso et al., 2022b).

Based on the well-known pro-inflammatory consequences of IL-6 systemically and within the CNS, we hypothesized that the perinatal increase in IL-6 would activate the resident immune cells of the CNS or stimulate infiltration of circulating leukocytes. Monocyte infiltration and microglial activation have been observed in MIA models induced by PolyI:C (Juckel et al., 2011) or LPS (Diz-Chaves et al., 2013; Smith et al., 2014), models of maternal allergic asthma using particulate ovalbumin (Tamayo et al., 2023) and a dual hit MIA model (Chen et al., 2020). Activating either TLR2 from P3-P8 in mice using the TLR2 agonist Pam (3)CSK or activating TLR4 using LPS elevated levels of IL-1β, IL-6, and the chemokines MCP-1 and KC. This was accompanied by strong microglial activation, especially with the TLR2 agonist (Du et al., 2011). These microglial changes have been suggested to contribute to ASD by changing the fates of SVZ-specific neural progenitors and by altering synaptic pruning (Bilbo et al., 2018; Loayza et al., 2023). However, we did not observe changes in microglia morphology or expression of CD68.

To determine whether immune cells might contribute to the aberrant behaviors described here, we compared the numbers of eight different immune cell types by flow cytometry between the brains of IL-6-treated mice and controls. To our surprise, there were no significant changes in the resident or infiltrating immune cells acutely after the IL-6 elevation. Our initial prediction was that there would be a detectable change in microglial activation, as microglia are particularly sensitive to IL-6, which stimulates STAT3 phosphorylation to switch them into a reactive state associated with phagocytosis (Couch et al., 2023; Lin and Levison, 2009; West et al., 2022). However, our flow cytometry studies revealed no differences in the number of reactive microglia following IL-6 Rx. Furthermore, there was no difference in microglial number, reactivity, or branching complexity in the frontal cortex at the same time point as the CNS IL-6 elevation. The late administration of IL-6 could explain why we didn’t see an effect on the microglia in this study. Ozaki et al., 2020 showed that MIA in mid-pregnancy increased IL-6 expression in embryonic microglia but failed to cause any marked changes in morphology either at E18 or postnatally. In contrast, earlier activation caused morphological changes in the microglial process and sustained behavioral deficits (Ozaki et al., 2020). As discussed above, the dose of IL-6 administered, which causes a mild increase in CNS levels of IL-6, might also explain why we are not observing increased microglia activation.

IL-6 is known to recruit immune cells to sites of inflammation along with other pro-inflammatory cytokines such as IL-1β and TNFα. These cytokines, when released by tissue-resident immune cells recruit immature Ly6c-high monocytes and neutrophils to infected or injured tissues (Hodes et al., 2015; Rosenblat et al., 2014). Systemic activation of TLR2 (but not TLR4) in mice from P3-P8 stimulates neutrophil and monocyte infiltration into the CSF and subsequently into periventricular regions of the brain from the choroid plexus (Mottahedin et al., 2019; Mottahedin et al., 2017). Therefore, we assessed the levels of macrophages, monocytes and Ly6c + neutrophils in our model, but again found no differences in IL-6 treated mice compared to controls. We also found no differences in the numbers of mast cells, T-cells, and dendritic cells, which agrees with the fact that we did not observe any increase of chemokines in the CNS and further support the conclusion that IL-6 is not promoting neuroinflammation in this model. This might indicate that the elevated body temperature in treated mice is a direct effect of IL-6 acting as a pyrogen (Opp et al., 1989; Rothwell et al., 1991) by stimulating the production of prostaglandin E2 (Dinarello et al., 1991) via STAT3 in the hypothalamus (Thomas Hübschle et al., 2001).

While our analyses of neuroinflammation did not support our predictions, our studies clearly showed that the behavioral changes elicited by the IL-6 treatment can be detected very early. Here, we show that alterations in communication, social interactions, and repetitive behaviors can all be detected within the first 3 weeks of life. The earliest change that we could detect was in ultrasonic vocalizations. Young rodents cannot maintain their body temperature for more than a few minutes outside the nest, succumbing to hypothermic death. So, when a young pup leaves the nest, it makes urgent USV calls to elicit maternal retrieval. In our USV experiments, IL-6-treated P7 male, but not female pups produced more calls during isolation from the nest. The increased vocalizations were specifically of short, less complex morphology. This contrasts with most MIA studies that use PolyI:C or LPS at midgestation, where the male offspring produce fewer isolation vocalizations at the second postnatal week (Carbone et al., 2023; Kirsten et al., 2012; Malkova et al., 2012; Martz et al., 2023; Pendyala et al., 2017; Usui et al., 2022). The contrast between our data and the MIA literature is possibly due to the time of injections. Notably, when Carlezon et al., 2019 compared prenatal PolyI:C to postnatal LPS treatments, they found that the prenatal treatment reduced the number of USVs only in male pups at the second postnatal week, while postnatal treatment led to an increase in USVs in both males and females at the same age (Carlezon et al., 2019). Interestingly, female IL-6-injected pups in our study had the same number and morphology of calls but used lower-frequency vocalizations. Despite sex differences in USVs, male and female IL-6-injected pups were retrieved more slowly by the dam than PBS-injected pups. This suggests that the dam is detecting different vocalizations from the IL-6 Rx males and females but that the alterations that she is detecting differ between the sexes. Alternatively, there may be other differences that we did not measure that are affecting maternal retrieval. Regardless, this finding suggests that maternal care is contributing to some of the behavior phenotypes described here, which will need to be investigated further in future studies.

For sociability, our goal was to detect deficits as early as possible. At P21, fine-grained measures of interactions between pairs of juvenile mice placed together in standard cages provide the most detailed insights into reciprocal social interactions (McFarlane et al., 2008; Silverman et al., 2010). Maternal Immune activation with either PolyI:C or LPS unambiguously leads to deficits in sociability in young male mice at P35 (Carbone et al., 2023), P30 (Martz et al., 2023) (LPS) (Vuillermot et al., 2017) (PolyI:C), and as early as P25 (Oskvig et al., 2012). However, only Carbone et al., 2023 showed deficits in social play, while others have used the 3-chamber social approach test, which is less reliable at that age (Carbone et al., 2023). Our approach allowed us to show a very early and robust deficit in juvenile play at P21. At the same age, we also detected increased self-grooming, an analog of the repetitive behaviors observed in ASD and other neurodevelopmental disorders and a common finding in MIA models, where mice spend as much as 50 % more time grooming than controls (Malkova et al., 2012). However, those studies typically evaluate grooming at later ages (Lan et al., 2023; Malkova et al., 2012). Increased self-grooming at P21 has only been observed in genetic models of ASD (McFarlane et al., 2008). Here, self-grooming was more robust in the pre-pubescent IL-6-injected male mice, showing that this phenotype emerges later in the females, as both post-pubescent male and female IL-6 Rx mice exhibited increased self-grooming (Velloso et al., 2022b).

Finally, we detected hyper-mechanosensitivity as early as P13, reproducing what is observed mainly at later ages for genetic mouse models of ASD, including Shank3, Fmr1 and Mecp2 (Schaffler et al., 2019) and corresponds with the high prevalence of tactile hypersensitivity in children with attention-deficit/hyperactivity disorder (ADHD) (He et al., 2021) and ASD (Puts et al., 2014; Tomchek and Dunn, 2007). Tactile Hypersensitivity in ASD models is correlated with decreased Parvalbumin + interneurons in the somatosensory cortex (Deemyad et al., 2022), and higher maternal IL-6 is associated with stronger connectivity between the amygdala and the somatosensory cortex (Graham et al., 2018), suggesting that IL-6 might affect synaptogenesis in brain regions involved in sensory processing and integration.

Taken altogether, our new data indicate that the effects of the IL-6 treatment on behavior are not a consequence of downstream neuroinflammation. We had previously shown that IL-6 alters the proliferation and subsequent fates of stem cells and progenitors from the secondary germinal matrices of the brain and had concluded that the subsequent alteration in the cellular composition of the brain was contributing to the behavioral changes elicited by the IL-6 Rx. Additionally, CNS neurons express IL-6Rα and gp130 (Hampel et al., 2005) in pre-and post-synaptic membranes (D’Arcangelo et al., 2000) and IL-6 overexpression leads to progressive synaptic damage and loss of inhibitory interneurons (Heyser et al., 1997). Collectively, *in vivo* and *in vitro* studies have established that IL-6 has neuronal and synaptic actions that are evident at multiple levels, including protein expression, signal transduction, neuronal function, synaptic mechanisms and synaptic function (Gruol, 2015). These studies provide additional insights into mechanisms responsible for the observed early changes in behavior. Our ongoing studies are assessing possible mechanisms responsible for the persistence of the observed behaviors, including evaluating altered neurogenesis and gliogenesis, synaptic transmission and altered perineuronal nets.

## Supplementary Material

1

2

## Figures and Tables

**Table 1:** Antibodies used for Flow Cytometry to define immune cell types.

Marker	Fluorophore	Isotype	Clone	Host	Target	Company	Catalogue
CD45	SuperBright 600	IgG2b k	30-F11	Rat	Mouse	Thermo Fisher	63-0451-82
CD11b	SuperBright 436	IgG2b k	M1/70	Rat	Mouse	Thermo Fisher	62-0112-82
Ly-6G	SuperBright 702	IgG2a k	1A8-Ly6g	Rat	Mouse	Thermo Fisher	67-9668-80
Ly-6C	Alexa Fluor 488	IgG2c k	HK1.4	Rat	Mouse	Thermo Fisher	53-5932-80
CD3	SuperBright 780	IgG2b k	17A2	Rat	Mouse	Thermo Fisher	78-0032-82
CD4	PE-Cy5	IgG2b k	GK1.5	Rat	Mouse	Thermo Fisher	15-0041-81
CD117	PE	IgG2b k	ACK2	Rat	Mouse	Thermo Fisher	12-1172-83
F4/80	PE-Cy7	IgG2a k	BM8	Rat	Mouse	Thermo Fisher	25-4801-82

**Table 2:** Primers used for RT-qPCR.

Target	Company	Product Name	GeneGlobe ID / Cat#	Transcripts detected
IL-6	Qiagen	Mm_Il6_1_SG QuantiTect Primer Assay	QT00098875	NM_031168
IL-10	Qiagen	Mm_Il10_1_SG QuantiTect Primer Assay	QT00106169	NM_010548
IL-13	Qiagen	Mm_Il13_1_SG QuantiTect Primer Assay	QT00099554	NM_008355
IL-4	Qiagen	Mm_Il4_1_SG QuantiTect Primer Assay	QT00160678	NM_021283
IL-1a	Qiagen	Mm_Il1a_1_SG QuantiTect Primer Assay	QT00113505	NM_010554 XM_006498794
LIF	Qiagen	Mm_Lif_va.1_SG QuantiTect Primer Assay	QT01161685	NM_008501
				XM_006514542
				XM_006514543XM_006514544
IL-17a	Qiagen	Mm_il17a_1_SG Quantitect Primer Assay	QT00103278	NM_010552
PPia	IDT	Mm.PT.39a.2.gs		NM_008907(1)

**Table 3:** Mrna expression levels of il-6, il-10 and il-13 in the frontal cortex of p7 pbs and il-6-injected mice.

Cytokine	Treatment	All mice	Males	Females
IL-6	PBS	1.00 +/− 0.18	0.82 +/− 0.21	1.18 +/− 0.15
	IL-6	1.07 +/− 0.09	1.62 +/− 0.19	0.98 +/− 0.08
IL-10	PBS	1.00 +/− 0.46	0.54 +/− 0.34	1.46 +/− 0.42
	IL-6	1.57 +/− 0.37	1.93 +/− 0.66	1.19 +/− 0.65
IL-13*	PBS	1.00 +/− 0.35	0.65 +/− 0.19	1.35 +/− 0.32
	IL-6	1.13 +/− 0.36	0.77 +/− 0.46	1.48 +/− 0.41
IL-17a	PBS	1.00 +/− 0.28	1.00 +/− 0.34	1.00 +/− 0.25
	IL-6	0.71 +/− 0.30	0.82 +/− 0.34	0.60 +/− 0.28

## Data Availability

Data will be made available on request.

## Appendix A. Supplementary data

Supplementary data to this article can be found online at https://doi.org/10.1016/j.bbi.2025.02.009.

## References

[R1] Aaltonen R, Heikkinen T, Hakala K, Laine K, Alanen A, 2005. Transfer of proinflammatory cytokines across term placenta. Obstet. Gynecol 106, 802–807. 10.1097/01.AOG.0000178750.84837.ed. 16199639

[R2] Abdallah MW, Larsen N, Grove J, Norgaard-Pedersen B, Thorsen P, Mortensen EL, Hougaard DM, 2013. Amniotic fluid inflammatory cytokines: potential markers of immunologic dysfunction in autism spectrum disorders. World J Biol Psychiatry 14, 528–538 22175527 10.3109/15622975.2011.639803

[R3] American Psychiatric Association, 2013. Diagnostic and statistical manual of mental disorders, 5th ed. American Psychiatric Association.

[R4] Amgalan A, Andescavage N, Limperopoulos C, 2021. Prenatal origins of neuropsychiatric diseases. Acta Paediatr. 10.1111/apa.15766. 33475192

[R5] Ashwood P, Krakowiak P, Hertz-Picciotto I, Hansen R, Pessah I, Van de Water J, 2011. Elevated plasma cytokines in autism spectrum disorders provide evidence of immune dysfunction and are associated with impaired behavioral outcome. Brain Behav. Immun 25, 40–45. 10.1016/j.bbi.2010.08.003. 20705131 PMC2991432

[R6] Banks WA, Kastin AJ, Broadwell RD, 1995. Passage of cytokines across the blood-brain barrier. Neuroimmunomodulation 2, 241–248. 10.1159/000097202. 8963753

[R7] Banks WA, Kastin AJ, Gutierrez EG, 1994. Penetration of interleukin-6 across the murine blood-brain barrier. Neurosci. Lett 179, 53–56. 7845624 10.1016/0304-3940(94)90933-4

[R8] Barron JJ, Mroz NM, Taloma SE, Dahlgren MW, Ortiz-Carpena J, Dorman LC, Vainchtein ID, Escoubas CC, Molofsky AB, Molofsky AV, 2023. Group 2 innate lymphoid cells promote inhibitory synapse development and social behavior. bioRxiv 10.1101/2023.03.16.532850. PMC11995778 39480923

[R9] Bear JJ, Wu YW, 2016. Maternal Infections During Pregnancy and Cerebral Palsy in the Child. Pediatr. Neurol 57, 74–79. 10.1016/j.pediatrneurol.2015.12.018. 26857522 PMC4801683

[R10] Bilbo SD, Block CL, Bolton JL, Hanamsagar R, Tran PK, 2018. Beyond infection - Maternal immune activation by environmental factors, microglial development, and relevance for autism spectrum disorders. Exp. Neurol 299, 241–251. 10.1016/j.expneurol.2017.07.002. 28698032 PMC5723548

[R11] Carbone E, Buzzelli V, Manduca A, Leone S, Rava A, Trezza V, 2023. Maternal Immune Activation Induced by Prenatal Lipopolysaccharide Exposure Leads to Long-Lasting Autistic-like Social, Cognitive and Immune Alterations in Male Wistar Rats. Int. J. Mol. Sci 24. 10.3390/ijms24043920. PMC9968168 36835329

[R12] Carlezon WA Jr., Kim W, Missig G, Finger BC, Landino SM, Alexander AJ, Mokler EL, Robbins JO, Li Y, Bolshakov VY, McDougle CJ, Kim KS, 2019. Maternal and early postnatal immune activation produce sex-specific effects on autism-like behaviors and neuroimmune function in mice. Sci. Rep 9, 16928. 10.1038/s41598-019-53294-z. 31729416 PMC6858355

[R13] Carter CJ, Blizard RA, 2016. Autism genes are selectively targeted by environmental pollutants including pesticides, heavy metals, bisphenol A, phthalates and many others in food, cosmetics or household products. Neurochem. Int 10.1016/j.neuint.2016.10.011. 27984170

[R14] Caruso A, Marconi MA, Scattoni ML, Ricceri L, 2022. Ultrasonic vocalizations in laboratory mice: strain, age, and sex differences. Genes Brain Behav. 21, e12815. 10.1111/gbb.12815. 35689354 PMC9744514

[R15] Caruso A, Ricceri L, Scattoni ML, 2020. Ultrasonic vocalizations as a fundamental tool for early and adult behavioral phenotyping of Autism Spectrum Disorder rodent models. Neurosci. Biobehav. Rev 116, 31–43. 10.1016/j.neubiorev.2020.06.011. 32544538

[R16] Chamera K, Kotarska K, Szuster-Głuszczak M, Trojan E, Skórkowska A, Pomierny B, Krzyżanowska W, Bryniarska N, Basta-Kaim A, 2020. The prenatal challenge with lipopolysaccharide and polyinosinic:polycytidylic acid disrupts CX3CL1-CX3CR1 and CD200-CD200R signalling in the brains of male rat offspring: a link to schizophrenia-like behaviours. J. Neuroinflammation 17, 247. 10.1186/s12974-020-01923-0. 32829711 PMC7444338

[R17] Chen HR, Chen CW, Mandhani N, Short-Miller JC, Smucker MR, Sun YY, Kuan CY, 2020. Monocytic Infiltrates Contribute to Autistic-like Behaviors in a Two-Hit Model of Neurodevelopmental Defects. J. Neurosci 40, 9386–9400. 10.1523/jneurosci.1171-20.2020. 33127853 PMC7724142

[R18] Choi SS, Lee HJ, Lim I, Satoh J, Kim SU, 2014. Human astrocytes: secretome profiles of cytokines and chemokines. PLoS One 9, e92325. 10.1371/journal.pone.0092325. 24691121 PMC3972155

[R19] Coffey KR, Marx RE, Neumaier JF, 2019. DeepSqueak: a deep learning-based system for detection and analysis of ultrasonic vocalizations. Neuropsychopharmacology 44, 859–868. 10.1038/s41386-018-0303-6. 30610191 PMC6461910

[R20] Couch A, Solomon S, R RRD, Marrocu A, Sun Y, Sichlinger L, Matuleviciute R, Dutan Polit L, Hanger B, Brown A, Kordasti S, Srivastava DP, Vernon AC, 2023. Acute IL-6 exposure triggers canonical IL6Ra signaling in hiPSC microglia, but not neural progenitor cells. Brain Behav. Immun 10.1016/j.bbi.2023.02.007. PMC10682389 36781081

[R21] D’Arcangelo G, Tancredi V, Onofri F, D’Antuono M, Giovedi S, Benfenati F, 2000. Interleukin-6 inhibits neurotransmitter release and the spread of excitation in the rat cerebral cortex. Eur. J. Neurosci 12, 1241–1252. 10.1046/j.1460-9568.2000.00011.x. 10762353

[R22] Dahlgren J, Samuelsson AM, Jansson T, Holmang A, 2006. Interleukin-6 in the maternal circulation reaches the rat fetus in mid-gestation. Pediatr. Res 60, 147–151. 10.1203/01.pdr.0000230026.74139.18. 16864694

[R23] Dawson MS, Gordon-Fleet K, Yan L, Tardos V, He H, Mui K, Nawani S, Asgarian Z, Catani M, Fernandes C, Drescher U, 2023. Sexual dimorphism in the social behaviour of Cntnap2-null mice correlates with disrupted synaptic connectivity and increased microglial activity in the anterior cingulate cortex. Commun. Biol 6, 846. 10.1038/s42003-023-05215-0. 37582968 PMC10427688

[R24] Deemyad T, Puig S, Papale AE, Qi H, LaRocca GM, Aravind D, LaNoce E, Urban NN, 2022. Lateralized Decrease of Parvalbumin+ Cells in the Somatosensory Cortex of ASD Models Is Correlated with Unilateral Tactile Hypersensitivity. Cereb. Cortex 32, 554–568. 10.1093/cercor/bhab233. 34347040 PMC8805834

[R25] Desbonnet L, Konkoth A, Laighneach A, McKernan D, Holleran L, McDonald C, Morris DW, Donohoe G, Kelly J, 2022. Dual hit mouse model to examine the long-term effects of maternal immune activation and post-weaning social isolation on schizophrenia endophenotypes. Behav. Brain Res 430, 113930. 10.1016/j.bbr.2022.113930. 35609792

[R26] Dinarello CA, Cannon JG, Mancilla J, Bishai I, Lees J, Coceani F, 1991. Interleukin-6 as an endogenous pyrogen: induction of prostaglandin E2 in brain but not in peripheral blood mononuclear cells. Brain Res. 562, 199–206. 10.1016/0006-8993(91)90622-3. 1773338

[R27] Diz-Chaves Y, Astiz M, Bellini MJ, Garcia-Segura LM, 2013. Prenatal stress increases the expression of proinflammatory cytokines and exacerbates the inflammatory response to LPS in the hippocampal formation of adult male mice. Brain Behav. Immun 28, 196–206. 10.1016/j.bbi.2012.11.013. 23207108

[R28] Du X, Fleiss B, Li H, D’Angelo B, Sun Y, Zhu C, Hagberg H, Levy O, Mallard C, Wang X, 2011. Systemic stimulation of TLR2 impairs neonatal mouse brain development. PLoS One 6, e19583. 10.1371/journal.pone.0019583. 21573120 PMC3089625

[R29] Dutta S, Sengupta P, 2016. Men and mice: Relating their ages. Life Sci. 152, 244–248. 10.1016/j.lfs.2015.10.025. 26596563

[R30] Elwood RW, Keeling F, 1982. Temporal organization of ultrasonic vocalizations in infant mice. Dev. Psychobiol 15, 221–227. 10.1002/dev.420150306. 7095288

[R31] Garay PA, Hsiao EY, Patterson PH, McAllister AK, 2013. Maternal immune activation causes age- and region-specific changes in brain cytokines in offspring throughout development. Brain Behav. Immun 31, 54–68. 10.1016/j.bbi.2012.07.008. 22841693 PMC3529133

[R32] Graham AM, Rasmussen JM, Rudolph MD, Heim CM, Gilmore JH, Styner M, Potkin SG, Entringer S, Wadhwa PD, Fair DA, Buss C, 2018. Maternal Systemic Interleukin-6 During Pregnancy Is Associated With Newborn Amygdala Phenotypes and Subsequent Behavior at 2 Years of Age. Biol. Psychiatry 83, 109–119. 10.1016/j.biopsych.2017.05.027. 28754515 PMC5723539

[R33] Gruol DL, 2015. IL-6 regulation of synaptic function in the CNS. Neuropharmacology 96, 42–54. 10.1016/j.neuropharm.2014.10.023. 25445486 PMC4446251

[R34] Hampel H, Haslinger A, Scheloske M, Padberg F, Fischer P, Unger J, Teipel SJ, Neumann M, Rosenberg C, Oshida R, Hulette C, Pongratz D, Ewers M, Kretzschmar HA, Moller HJ, 2005. Pattern of interleukin-6 receptor complex immunoreactivity between cortical regions of rapid autopsy normal and Alzheimer’s disease brain. Eur. Arch. Psychiatry Clin. Neurosci 255, 269–278. 10.1007/s00406-004-0558-2. 15565298

[R35] Han VX, Patel S, Jones HF, Nielsen TC, Mohammad SS, Hofer MJ, Gold W, Brilot F, Lain SJ, Nassar N, Dale RC, 2021. Maternal acute and chronic inflammation in pregnancy is associated with common neurodevelopmental disorders: a systematic review. Transl. Psychiatry 11, 71. 10.1038/s41398-021-01198-w. 33479207 PMC7820474

[R36] He JL, Wodka E, Tommerdahl M, Edden RAE, Mikkelsen M, Mostofsky SH, Puts NAJ, 2021. Disorder-specific alterations of tactile sensitivity in neurodevelopmental disorders. Commun. Biol 4, 97. 10.1038/s42003-020-01592-y. 33483581 PMC7822903

[R37] Heyser CJ, Masliah E, Samimi A, Campbell IL, Gold LH, 1997. Progressive decline in avoidance learning paralleled by inflammatory neurodegeneration in transgenic mice expressing interleukin 6 in the brain. Proc Natl Acad Sci U S A 94, 1500–1505. 10.1073/pnas.94.4.1500. 9037082 PMC19820

[R38] Hida H, Mouri A, Ando Y, Mori K, Mamiya T, Iwamoto K, Ozaki N, Yamada K, Nabeshima T, Noda Y, 2014. Combination of neonatal PolyI:C and adolescent phencyclidine treatments is required to induce behavioral abnormalities with overexpression of GLAST in adult mice. Behav. Brain Res 258, 34–42. 10.1016/j.bbr.2013.09.026. 24060653

[R39] Hodes GE, Kana V, Menard C, Merad M, Russo SJ, 2015. Neuroimmune mechanisms of depression. Nat. Neurosci 18, 1386–1393. 10.1038/nn.4113. 26404713 PMC4843114

[R40] Hodyl NA, Krivanek KM, Lawrence E, Clifton VL, Hodgson DM, 2007. Prenatal exposure to a pro-inflammatory stimulus causes delays in the development of the innate immune response to LPS in the offspring. J. Neuroimmunol 190, 61–71. 10.1016/j.jneuroim.2007.07.021. 17804085

[R41] Hsiao EY, McBride SW, Chow J, Mazmanian SK, Patterson PH, 2012. Modeling an autism risk factor in mice leads to permanent immune dysregulation. Proc. Natl. Acad. Sci. U. S. A 109, 12776–12781. 10.1073/pnas.1202556109. 22802640 PMC3411999

[R42] Hsiao EY, Patterson PH, 2011. Activation of the maternal immune system induces endocrine changes in the placenta via IL-6. Brain Behav. Immun 25, 604–615. 10.1016/j.bbi.2010.12.017. 21195166 PMC3081363

[R43] Jiang HY, Xu LL, Shao L, Xia RM, Yu ZH, Ling ZX, Yang F, Deng M, Ruan B, 2016. Maternal infection during pregnancy and risk of autism spectrum disorders: A systematic review and meta-analysis. Brain Behav. Immun 58, 165–172. 10.1016/j.bbi.2016.06.005. 27287966

[R44] Jones KL, Croen LA, Yoshida CK, Heuer L, Hansen R, Zerbo O, DeLorenze GN, Kharrazi M, Yolken R, Ashwood P, Van de Water J, 2017. Autism with intellectual disability is associated with increased levels of maternal cytokines and chemokines during gestation. Mol. Psychiatry 22, 273–279. 10.1038/mp.2016.77. 27217154 PMC5122473

[R45] Juckel G, Manitz MP, Brüne M, Friebe A, Heneka MT, Wolf RJ, 2011. Microglial activation in a neuroinflammational animal model of schizophrenia–a pilot study. Schizophr. Res 131, 96–100. 10.1016/j.schres.2011.06.018. 21752601

[R46] Kirsten TB, Casarin RC, Bernardi MM, Felicio LF, 2018. Pioglitazone abolishes autistic-like behaviors via the IL-6 pathway. PLoS One 13, e0197060. 10.1371/journal.pone.0197060. 29791472 PMC5965820

[R47] Kirsten TB, Chaves-Kirsten GP, Chaible LM, Silva AC, Martins DO, Britto LR, Dagli ML, Torrao AS, Palermo-Neto J, Bernardi MM, 2012. Hypoactivity of the central dopaminergic system and autistic-like behavior induced by a single early prenatal exposure to lipopolysaccharide. J. Neurosci. Res 90, 1903–1912. 10.1002/jnr.23089. 22714803

[R48] Kumari E, Velloso FJ, Nasuhidehnavi A, Somasundaram A, Savanur VH, Buono KD, Levison SW, 2020. Developmental IL-6 Exposure Favors Production of PDGF-Responsive Multipotential Progenitors at the Expense of Neural Stem Cells and Other Progenitors. Stem Cell Rep. 14, 861–875. 10.1016/j.stemcr.2020.03.019. PMC7220986 32302560

[R49] Lacroix S, Chang L, Rose-John S, Tuszynski MH, 2002. Delivery of hyper-interleukin-6 to the injured spinal cord increases neutrophil and macrophage infiltration and inhibits axonal growth. J Comp Neurol 454, 213–228. 12442313 10.1002/cne.10407

[R50] Lan XY, Gu YY, Li MJ, Song TJ, Zhai FJ, Zhang Y, Zhan JS, Bockers TM, Yue XN, Wang JN, Yuan S, Jin MY, Xie YF, Dang WW, Hong HH, Guo ZR, Wang XW, Zhang R, 2023. Poly(I:C)-induced maternal immune activation causes elevated self-grooming in male rat offspring: Involvement of abnormal postpartum static nursing in dam. Front. Cell Dev. Biol 11, 1054381. 10.3389/fcell.2023.1054381. 37009477 PMC10062710

[R51] Li Y, Wang X, Yang H, Li Y, Gui J, Cui Y, 2022. Profiles of Proinflammatory Cytokines and T Cells in Patients With Tourette Syndrome: A Meta-Analysis. Front. Immunol 13, 843247. 10.3389/fimmu.2022.843247. 35693824 PMC9177955

[R52] Lin HW, Levison SW, 2009. Context-dependent IL-6 potentiation of interferon-gamma-induced IL-12 secretion and CD40 expression in murine microglia. J. Neurochem 111, 808–818. 10.1111/j.1471-4159.2009.06366.x. 19712053

[R53] Lipina TV, Zai C, Hlousek D, Roder JC, Wong AH, 2013. Maternal immune activation during gestation interacts with Disc1 point mutation to exacerbate schizophrenia-related behaviors in mice. J. Neurosci 33, 7654–7666. 10.1523/jneurosci.0091-13.2013. 23637159 PMC6618962

[R54] Loayza M, Lin S, Carter K, Ojeda N, Fan LW, Ramarao S, Bhatt A, Pang Y, 2023. Maternal immune activation alters fetal and neonatal microglia phenotype and disrupts neurogenesis in mice. Pediatr. Res 93, 1216–1225. 10.1038/s41390-022-02239-w. 35963885

[R55] Majerczyk D, Ayad EG, Brewton KL, Saing P, Hart PC, 2022. Systemic maternal inflammation promotes ASD via IL-6 and IFN-gamma. Biosci. Rep 42. 10.1042/BSR20220713. PMC9670245 36300375

[R56] Malkova NV, Yu CZ, Hsiao EY, Moore MJ, Patterson PH, 2012. Maternal immune activation yields offspring displaying mouse versions of the three core symptoms of autism. Brain Behav. Immun 26, 607–616. 10.1016/j.bbi.2012.01.011. 22310922 PMC3322300

[R57] Martz J, Shelton MA, Geist L, Seney ML, Kentner AC, 2023. Sex differences in offspring risk and resilience following 11beta-hydroxylase antagonism in a rodent model of maternal immune activation. Neuropsychopharmacology. 10.1038/s41386-023-01771-5. PMC11109257 38007547

[R58] Marz P, Heese K, Dimitriades-Schmutz B, Rose-John S, Otten U, 1999. Role of interleukin-6 and soluble IL-6 receptor in region-specific induction of astrocytic differentiation and neurotrophin expression. Glia 26, 191–200. 10340760 10.1002/(sici)1098-1136(199905)26:3<191::aid-glia1>3.0.co;2-#

[R59] Matsuyama T, Salter BM, Emami Fard N, Machida K, Sehmi R, 2024. TNF Superfamily and ILC2 Activation in Asthma. Biomolecules 14. 10.3390/biom14030294. PMC10967788 38540714

[R60] McFarlane HG, Kusek GK, Yang M, Phoenix JL, Bolivar VJ, Crawley JN, 2008. Autism-like behavioral phenotypes in BTBR T+tf/J mice. Genes Brain Behav. 7, 152–163. 10.1111/j.1601-183X.2007.00330.x. 17559418

[R61] Mednick SA, Machon RA, Huttunen MO, Bonett D, 1988. Adult schizophrenia following prenatal exposure to an influenza epidemic. Arch. Gen. Psychiatry 45, 189–192. 3337616 10.1001/archpsyc.1988.01800260109013

[R62] Meyer U, Nyffeler M, Engler A, Urwyler A, Schedlowski M, Knuesel I, Yee BK, Feldon J, 2006. The time of prenatal immune challenge determines the specificity of inflammation-mediated brain and behavioral pathology. J. Neurosci 26, 4752–4762. 10.1523/JNEUROSCI.0099-06.2006. 16672647 PMC6674174

[R63] Misiak B, Wójta-Kempa M, Samochowiec J, Schiweck C, Aichholzer M, Reif A, Samochowiec A, Stańczykiewicz B, 2022. Peripheral blood inflammatory markers in patients with attention deficit/hyperactivity disorder (ADHD): A systematic review and meta-analysis. Prog. Neuropsychopharmacol. Biol. Psychiatry 118, 110581. 10.1016/j.pnpbp.2022.110581. 35660454

[R64] Mottahedin A, Joakim Ek C, Truve K, Hagberg H, Mallard C, 2019. Choroid plexus transcriptome and ultrastructure analysis reveals a TLR2-specific chemotaxis signature and cytoskeleton remodeling in leukocyte trafficking. Brain Behav. Immun 79, 216–227. 10.1016/j.bbi.2019.02.004. 30822467 PMC6591031

[R65] Mottahedin A, Smith PL, Hagberg H, Ek CJ, Mallard C, 2017. TLR2-mediated leukocyte trafficking to the developing brain. J. Leukoc. Biol 101, 297–305. 10.1189/jlb.3A1215-568R. 27493242

[R66] Mueller FS, Scarborough J, Schalbetter SM, Richetto J, Kim E, Couch A, Yee Y, Lerch JP, Vernon AC, Weber-Stadlbauer U, Meyer U, 2021. Behavioral, neuroanatomical, and molecular correlates of resilience and susceptibility to maternal immune activation. Mol. Psychiatry 26, 396–410. 10.1038/s41380-020-00952-8. 33230204 PMC7850974

[R67] Oberg HH, Wesch D, Grüssel S, Rose-John S, Kabelitz D, 2006. Differential expression of CD126 and CD130 mediates different STAT-3 phosphorylation in CD4 +CD25- and CD25high regulatory T cells. Int. Immunol 18, 555–563. 10.1093/intimm/dxh396. 16540526

[R68] Opp M, Obal F Jr., Cady AB, Johannsen L, Krueger JM, 1989. Interleukin-6 is pyrogenic but not somnogenic. Physiol. Behav 45, 1069–1072. 10.1016/0031-9384(89)90239-4. 2476835

[R69] Oskvig DB, Elkahloun AG, Johnson KR, Phillips TM, Herkenham M, 2012. Maternal immune activation by LPS selectively alters specific gene expression profiles of interneuron migration and oxidative stress in the fetus without triggering a fetal immune response. Brain Behav. Immun 26, 623–634. 10.1016/j.bbi.2012.01.015. 22310921 PMC3285385

[R70] Ozaki K, Kato D, Ikegami A, Hashimoto A, Sugio S, Guo Z, Shibushita M, Tatematsu T, Haruwaka K, Moorhouse AJ, Yamada H, Wake H, 2020. Maternal immune activation induces sustained changes in fetal microglia motility. Sci. Rep 10, 21378. 10.1038/s41598-020-78294-2. 33288794 PMC7721716

[R71] Patel S, Keating BA, Dale RC, 2022. Anti-inflammatory properties of commonly used psychiatric drugs. Front. Neurosci 16, 1039379. 10.3389/fnins.2022.1039379. 36704001 PMC9871790

[R72] Pendyala G, Chou S, Jung Y, Coiro P, Spartz E, Padmashri R, Li M, Dunaevsky A, 2017. Maternal Immune Activation Causes Behavioral Impairments and Altered Cerebellar Cytokine and Synaptic Protein Expression. Neuropsychopharmacology 42, 1435–1446. 10.1038/npp.2017.7. 28102228 PMC5436129

[R73] Puts NA, Wodka EL, Tommerdahl M, Mostofsky SH, Edden RA, 2014. Impaired tactile processing in children with autism spectrum disorder. J. Neurophysiol 111, 1803–1811. 10.1152/jn.00890.2013. 24523518 PMC4044368

[R74] Rasmussen JM, Graham AM, Entringer S, Gilmore JH, Styner M, Fair DA, Wadhwa PD, Buss C, 2019. Maternal Interleukin-6 concentration during pregnancy is associated with variation in frontolimbic white matter and cognitive development in early life. Neuroimage 185, 825–835. 10.1016/j.neuroimage.2018.04.020. 29654875 PMC6181792

[R75] Rose-John S, Waetzig GH, Scheller J, Grotzinger J, Seegert D, 2007. The IL-6/sIL-6R complex as a novel target for therapeutic approaches. Expert Opin. Ther. Targets 11, 613–624. 10.1517/14728222.11.5.613. 17465721

[R76] Rosenblat JD, Cha DS, Mansur RB, McIntyre RS, 2014. Inflamed moods: a review of the interactions between inflammation and mood disorders. Prog. Neuropsychopharmacol. Biol. Psychiatry 53, 23–34. 10.1016/j.pnpbp.2014.01.013. 24468642

[R77] Rothaug M, Becker-Pauly C, Rose-John S, 2016. The role of interleukin-6 signaling in nervous tissue. BBA 1863, 1218–1227. 10.1016/j.bbamcr.2016.03.018. 27016501

[R78] Rothwell NJ, Busbridge NJ, Lefeuvre RA, Hardwick AJ, Gauldie J, Hopkins SJ, 1991. Interleukin-6 is a centrally acting endogenous pyrogen in the rat. Can. J. Physiol. Pharmacol 69, 1465–1469. 10.1139/y91-219. 1777846

[R79] Rudolph MD, Graham AM, Feczko E, Miranda-Dominguez O, Rasmussen JM, Nardos R, Entringer S, Wadhwa PD, Buss C, Fair DA, 2018. Maternal IL-6 during pregnancy can be estimated from newborn brain connectivity and predicts future working memory in offspring. Nat. Neurosci 21, 765–772. 10.1038/s41593-018-0128-y. 29632361 PMC5920734

[R80] Samuelsson AM, Jennische E, Hansson HA, Holmang A, 2006. Prenatal exposure to interleukin-6 results in inflammatory neurodegeneration in hippocampus with NMDA/GABA(A) dysregulation and impaired spatial learning. Am. J. Physiol. Regul. Integr. Comp. Physiol 290, R1345–R1356. 10.1152/ajpregu.00268.2005. 16357100

[R81] Samuelsson AM, Ohrn I, Dahlgren J, Eriksson E, Angelin B, Folkow B, Holmang A, 2004. Prenatal exposure to interleukin-6 results in hypertension and increased hypothalamic-pituitary-adrenal axis activity in adult rats. Endocrinology 145, 4897–4911. 10.1210/en.2004-0742. 15284195

[R82] Schaffler MD, Middleton LJ, Abdus-Saboor I, 2019. Mechanisms of Tactile Sensory Phenotypes in Autism: Current Understanding and Future Directions for Research. Curr. Psychiatry Rep 21, 134. 10.1007/s11920-019-1122-0. 31807945 PMC6900204

[R83] Semple BD, Blomgren K, Gimlin K, Ferriero DM, Noble-Haeusslein LJ, 2013. Brain development in rodents and humans: Identifying benchmarks of maturation and vulnerability to injury across species. Prog. Neurobiol 106–107, 1–16. 10.1016/j.pneurobio.2013.04.001. PMC3737272 23583307

[R84] Shimizu Y, Tsukada T, Sakata-Haga H, Sakai D, Shoji H, Saikawa Y, Hatta T, 2021. Exposure to Maternal Immune Activation Causes Congenital Unfolded Protein Response Defects and Increases the Susceptibility to Postnatal Inflammatory Stimulation in Offspring. J. Inflamm. Res 14, 355–365. 10.2147/JIR.S294238. 33603435 PMC7886242

[R85] Silverman JL, Yang M, Lord C, Crawley JN, 2010. Behavioural phenotyping assays for mouse models of autism. Nat. Rev. Neurosci 11, 490–502. 10.1038/nrn2851. 20559336 PMC3087436

[R86] Smith PLP, Hagberg H, Naylor AS, Mallard C, 2014. Neonatal Peripheral Immune Challenge Activates Microglia and Inhibits Neurogenesis in the Developing Murine Hippocampus. Dev. Neurosci 36, 119–131. 24642725 10.1159/000359950

[R87] Smith SE, Li J, Garbett K, Mirnics K, Patterson PH, 2007. Maternal immune activation alters fetal brain development through interleukin-6. J. Neurosci 27, 10695–10702. 10.1523/JNEUROSCI.2178-07.2007. 17913903 PMC2387067

[R88] Sofroniew MV, 2014. Multiple roles for astrocytes as effectors of cytokines and inflammatory mediators. Neuroscientist 20, 160–172. 10.1177/1073858413504466. 24106265

[R89] Spann MN, Monk C, Scheinost D, Peterson BS, 2018. Maternal Immune Activation During the Third Trimester Is Associated with Neonatal Functional Connectivity of the Salience Network and Fetal to Toddler Behavior. J. Neurosci 38, 2877–2886. 10.1523/jneurosci.2272-17.2018. 29487127 PMC5852665

[R90] Stirling DR, Swain-Bowden MJ, Lucas AM, Carpenter AE, Cimini BA, Goodman A, 2021. CellProfiler 4: improvements in speed, utility and usability. BMC Bioinf. 22, 433. 10.1186/s12859-021-04344-9. PMC8431850 34507520

[R91] Tamayo JM, Osman HC, Schwartzer JJ, Pinkerton KE, Ashwood P, 2023. Characterizing the neuroimmune environment of offspring in a novel model of maternal allergic asthma and particulate matter exposure. J. Neuroinflammation 20, 252. 10.1186/s12974-023-02930-7. 37919762 PMC10621097

[R92] Thomas Hübschle E-M-H, Meyerhof W, Pehl U, Roth J, Gerstberger R, 2001. The central pyrogenic action of interleukin-6 is related to nuclear translocation of STAT3 in the anteroventral preoptic area of the rat brain. J. Therm. Biol 26, 299–305. 10.1016/S0306-4565(01)00034-1.

[R93] Threlkeld SW, Lynch JL, Lynch KM, Sadowska GB, Banks WA, Stonestreet BS, 2010. Ovine proinflammatory cytokines cross the murine blood-brain barrier by a common saturable transport mechanism. Neuroimmunomodulation 17, 405–410. 10.1159/000288265. 20516722 PMC2914440

[R94] Tomchek SD, Dunn W, 2007. Sensory processing in children with and without autism: a comparative study using the short sensory profile. Am J Occup Ther 61, 190–200. 10.5014/ajot.61.2.190. 17436841

[R95] Usui N, Matsumoto-Miyai K, Koyama Y, Kobayashi Y, Nakamura Y, Kobayashi H, Shimada S, 2022. Social Communication of Maternal Immune Activation-Affected Offspring Is Improved by Si-Based Hydrogen-Producing Agent. Front. Psych 13, 872302. 10.3389/fpsyt.2022.872302. PMC9043691 35492705

[R96] Valtcheva S, Issa HA, Bair-Marshall CJ, Martin KA, Jung K, Zhang Y, Kwon HB, Froemke RC, 2023. Neural circuitry for maternal oxytocin release induced by infant cries. Nature 621, 788–795. 10.1038/s41586-023-06540-4. 37730989 PMC10639004

[R97] Velloso FJ, Kumari E, Buono KD, Frondelli MJ, Levison SW, 2022a. Analyzing mouse neural stem cell and progenitor cell proliferation using EdU incorporation and multicolor flow cytometry. STAR Protoc. 3, 101065. 10.1016/j.xpro.2021.101065. 35005647 PMC8718722

[R98] Velloso FJ, Wadhwa A, Kumari E, Carcea I, Gunal O, Levison SW, 2022b. Modestly increasing systemic interleukin-6 perinatally disturbs secondary germinal zone neurogenesis and gliogenesis and produces sociability deficits. Brain Behav. Immun 101, 23–36. 10.1016/j.bbi.2021.12.015. 34954074 PMC8885860

[R99] Vuillermot S, Luan W, Meyer U, Eyles D, 2017. Vitamin D treatment during pregnancy prevents autism-related phenotypes in a mouse model of maternal immune activation. Mol. Autism 8, 9. 10.1186/s13229-017-0125-0. 28316773 PMC5351212

[R100] West PK, McCorkindale AN, Guennewig B, Ashhurst TM, Viengkhou B, Hayashida E, Jung SR, Butovsky O, Campbell IL, Hofer MJ, 2022. The cytokines interleukin-6 and interferon-alpha induce distinct microglia phenotypes. J. Neuroinflammation 19, 96. 10.1186/s12974-022-02441-x. 35429976 PMC9013466

[R101] Whitehouse CM, Lewis MH, 2015. Repetitive Behavior in Neurodevelopmental Disorders: Clinical and Translational Findings. Behav. Anal 38, 163–178. 10.1007/s40614-015-0029-2. 26543319 PMC4629512

[R102] Yang M, Bozdagi O, Scattoni ML, Wohr M, Roullet FI, Katz AM, Abrams DN, Kalikhman D, Simon H, Woldeyohannes L, Zhang JY, Harris MJ, Saxena R, Silverman JL, Buxbaum JD, Crawley JN, 2012. Reduced excitatory neurotransmission and mild autism-relevant phenotypes in adolescent Shank3 null mutant mice. J. Neurosci 32, 6525–6541. 10.1523/JNEUROSCI.6107-11.2012. 22573675 PMC3362928

[R103] Zarate-Lopez D, Garzon-Partida AP, Gonzalez-Perez O, Galvez-Contreras AY, 2024. Sex differences in autism-like behavior and dopaminergic neurons in substantia nigra of juvenile mice prenatally exposed to valproate. Dev. Psychobiol 66, e22469. 10.1002/dev.22469. 38351305

[R104] Zaretsky MV, Alexander JM, Byrd W, Bawdon RE, 2004. Transfer of inflammatory cytokines across the placenta. Obstetr. Gynecol 103, 546–550. 10.1097/01.AOG.0000114980.40445.83. 14990420

[R105] Zerbo O, Qian Y, Yoshida C, Grether JK, Van de Water J, Croen LA, 2015. Maternal Infection During Pregnancy and Autism Spectrum Disorders. J. Autism Dev. Disord 45, 4015–4025. 10.1007/s10803-013-2016-3. 24366406 PMC4108569

[R106] Zhang Q, Wang J, Deng F, Yan Z, Xia Y, Wang Z, Ye J, Deng Y, Zhang Z, Qiao M, Li R, Denduluri SK, Wei Q, Zhao L, Lu S, Wang X, Tang S, Liu H, Luu HH, Haydon RC, He TC, Jiang L, 2015. TqPCR: A Touchdown qPCR Assay with Significantly Improved Detection Sensitivity and Amplification Efficiency of SYBR Green qPCR. PLoS One 10, e0132666. 10.1371/journal.pone.0132666. 26172450 PMC4501803

[R107] Zhang X, Ibi M, Haga R, Iwata K, Matsumoto M, Asaoka N, Liu J, Katsuyama M, Yabe-Nishimura C, 2021. NOX1/NADPH oxidase affects the development of autism-like behaviors in a maternal immune activation model. Biochem Biophys Res Commun 534, 59–66. 10.1016/j.bbrc.2020.11.070. 33310189

