# Supplementary material for: Interleukin-6 produces behavioral deficits in pre-pubescent mice independent of neuroinflammation
Source: Brain Behav Immun. Author manuscript; Available in PMC 2026 May 20. (PMC13188840; doi:10.1016/j.bbi.2025.02.009)
Supplement: 1 [file NIHMS2171783-supplement-1.pdf]

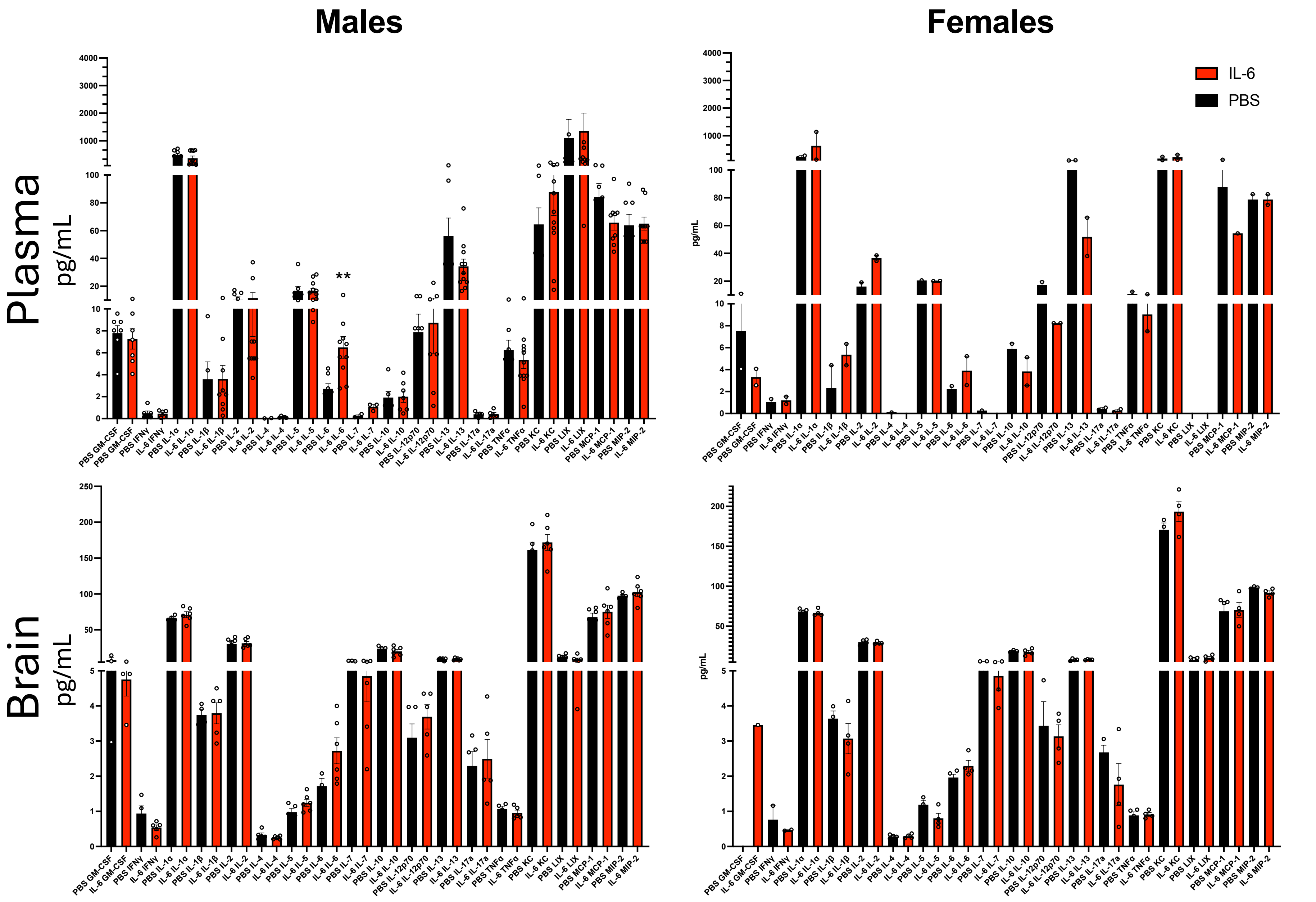

Fig.S1. Circulating and CNS levels of cytokines and chemokines in mice exposed to IL-6 postnatally

Acute circulating and CNS cytokine and chemokine levels (pg/mL) measured at P7 in the plasma and brain lysates of PBS and IL-6 male and female treated mice. ** P<0.01 by unpaired student t-test. n=19.

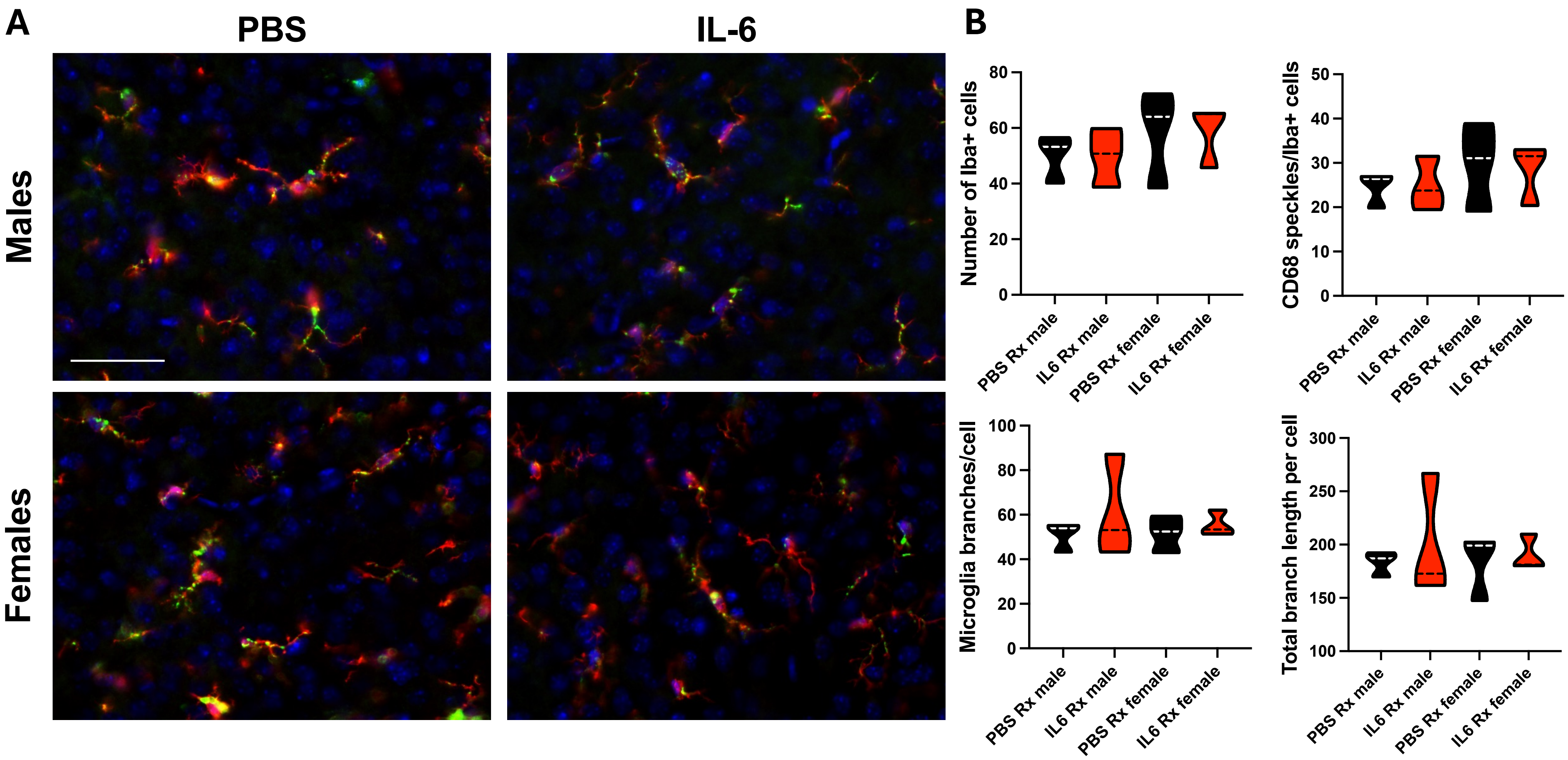

Fig.S2. IL-6 treatment does not increase acute microglia reactivity in the frontal cortex of male and female mice. (**A**) Microglia labelled for Iba1 (red) and CD68 (green) in the frontal cortex of P7 male and female mice. Nuclei labeled with DAPI. (**B**) Quantification of the number of Iba1+ cells, average CD68 speckles in Iba1+ cell, and number and length of microglia branches using CellProfiller. Scale bar = 50 μm. n=4 mice/group.

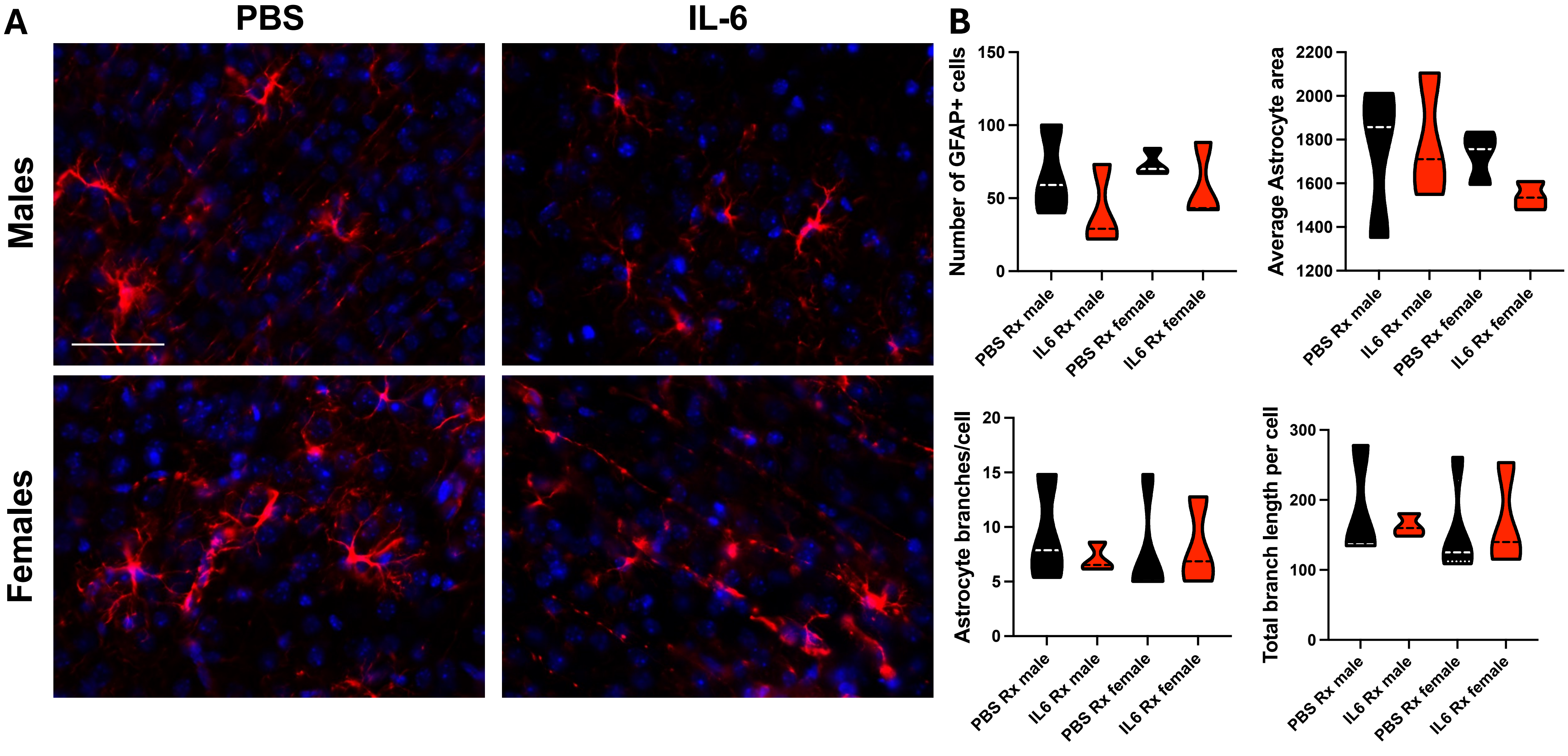

<u>Fig.S3. IL-6 treatment does not induce acute astrogliosis in the frontal cortex of male and female mice.</u>
(**A**) Astrocytes were labelled for GFAP (FG2.2; red) in the frontal cortex of P7 male and female mice. Nuclei labeled with DAPI. (**B**) Quantification of the number of GFAP+ cells, average area of cells, and number and length of astrocyte branches using CellProfiller. Scale bar = 50 µm. n=3 mice/group.
